# Supplementary figures and images for: Tempora: Cell trajectory inference using time-series single-cell RNA sequencing data
Source: PLoS Comput Biol. 2020 Sep 9;16(9):e1008205. doi: 10.1371/journal.pcbi.1008205 (PMC7505465; doi:10.1371/journal.pcbi.1008205)

**a**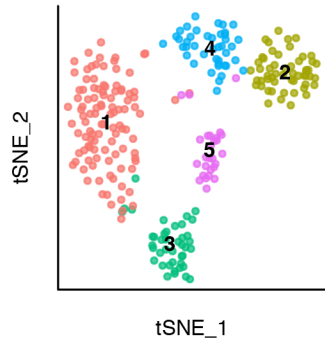**b**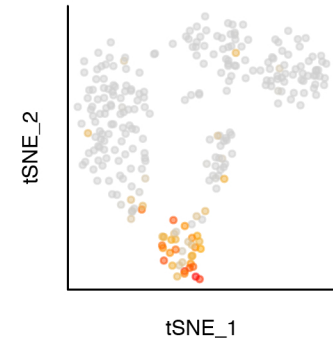**c**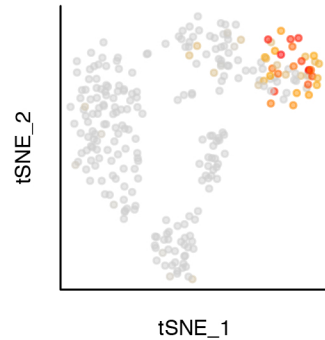**d**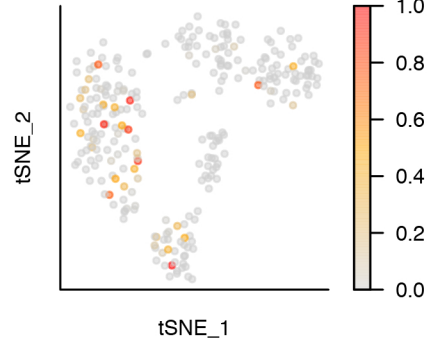

Supplement: S1 Fig — a. tSNE plot showing 271 cells in the HSMM data set, colored by cluster number. b-d. Visualization of known marker genes for b. myoblasts, c. myotubes and d. fibroblasts on the HSMM data set. (PDF) [file pcbi.1008205.s001.pdf]

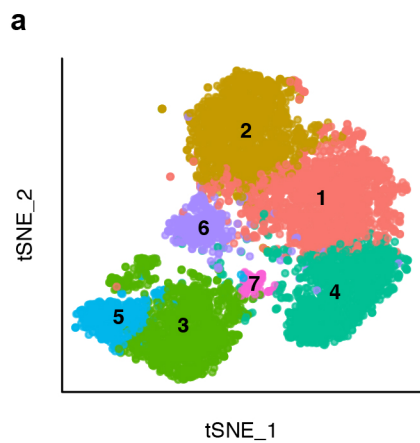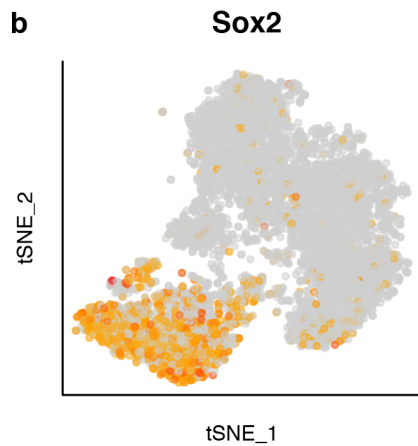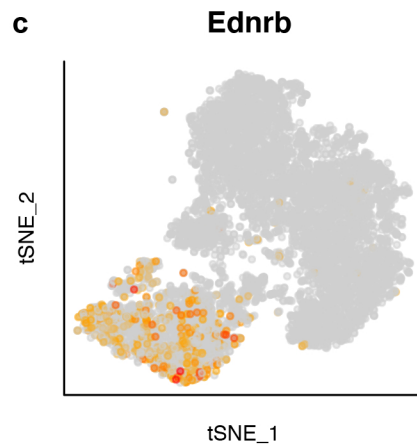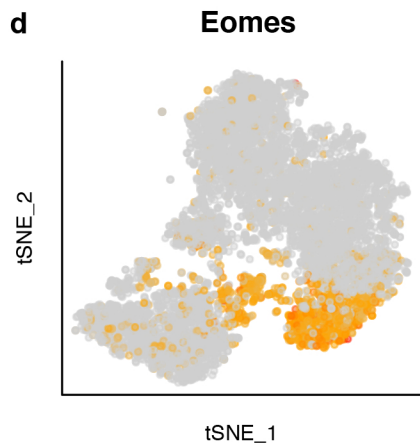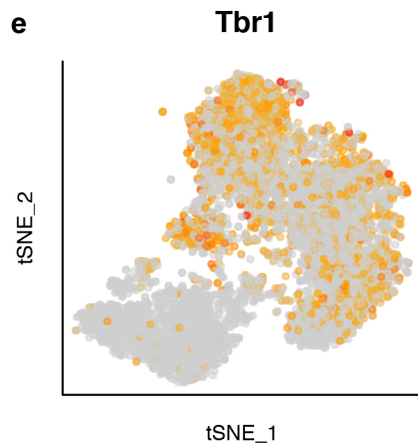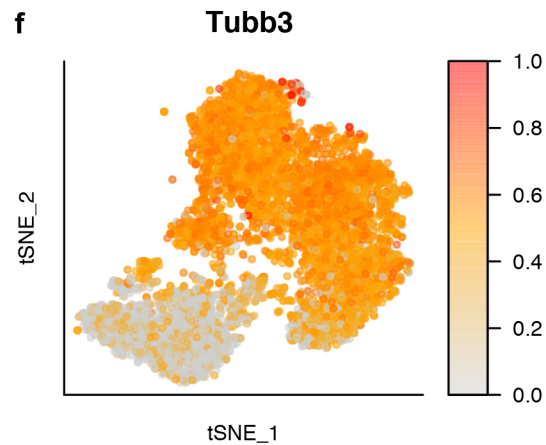

Supplement: S2 Fig — a. tSNE plot showing the ~6,000 neural cells captured in the murine cerebral cortex data set, colored by cluster number. b-f. Visualization of known marker genes for b. apical precursors (APs), c. radial precursors (RPs), d. cycling apical/radial precursors (AP/RPs), e. intermediate progenitors (IPs), f. early neurons and g. neurons in the murine cerebral cortex data set. (PDF) [file pcbi.1008205.s002.pdf]

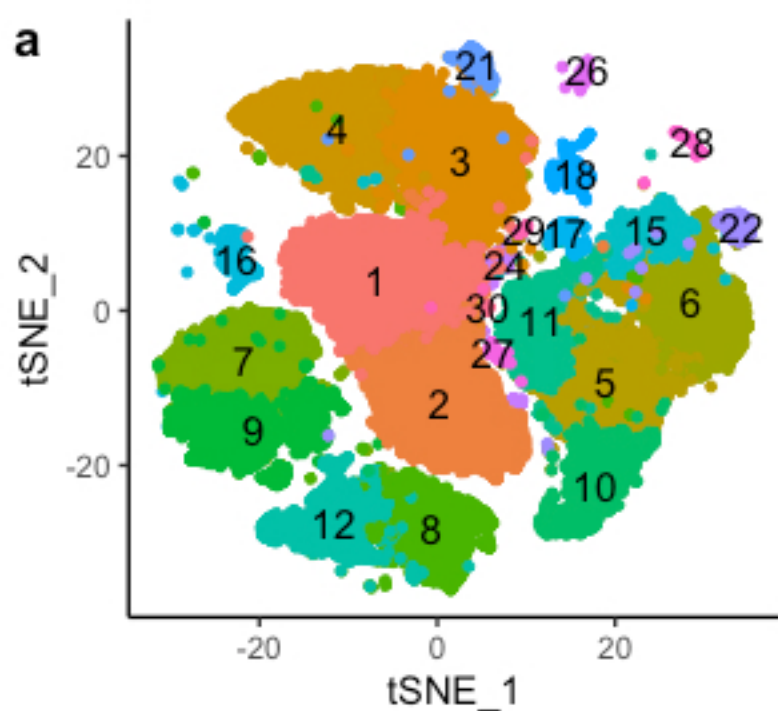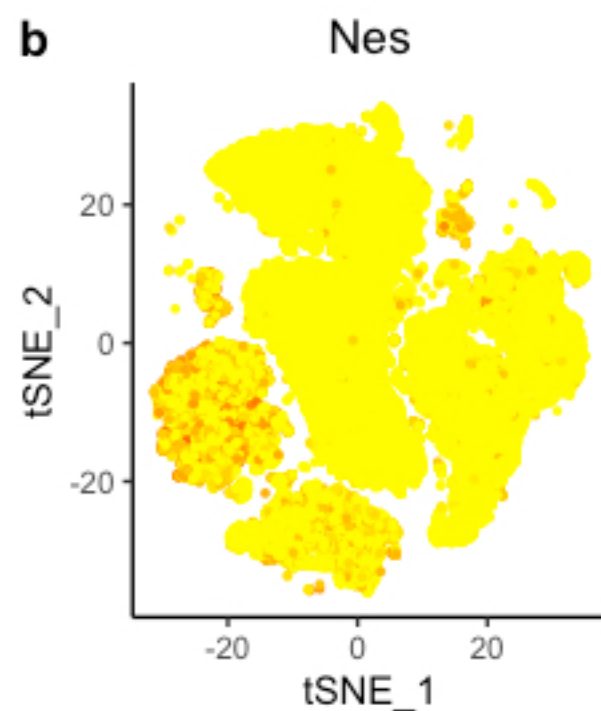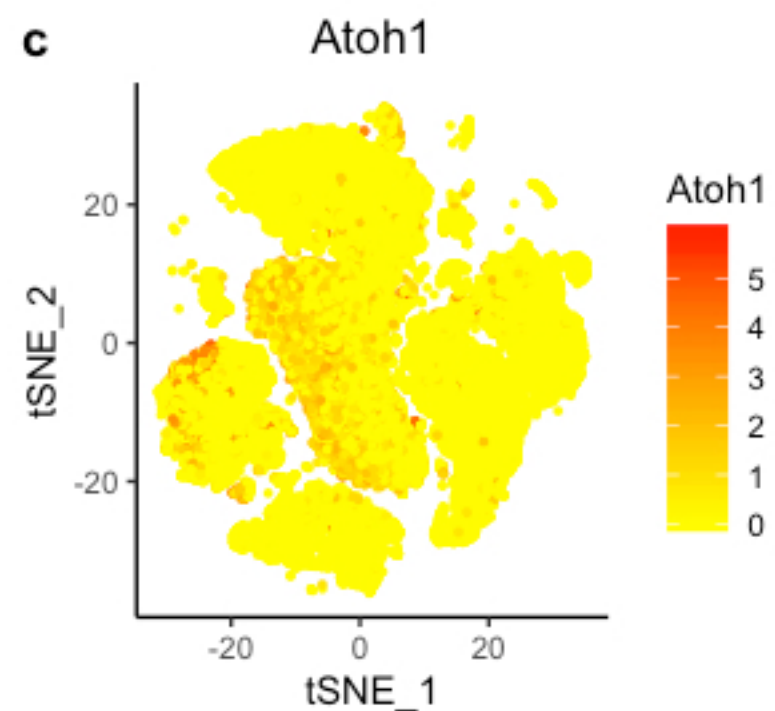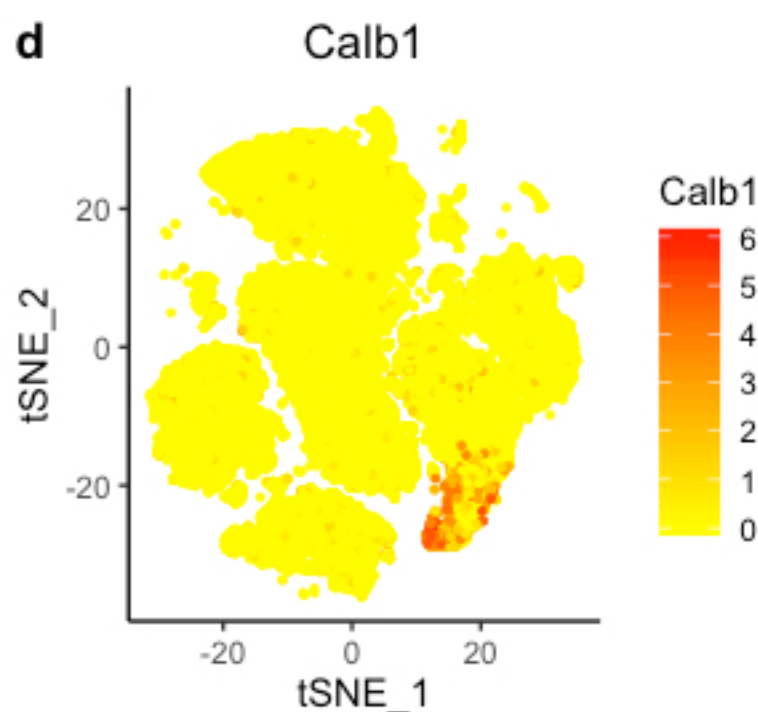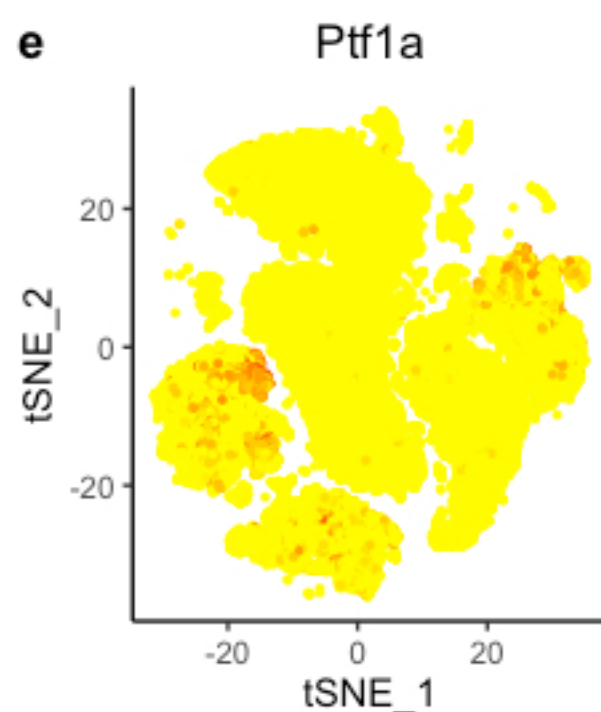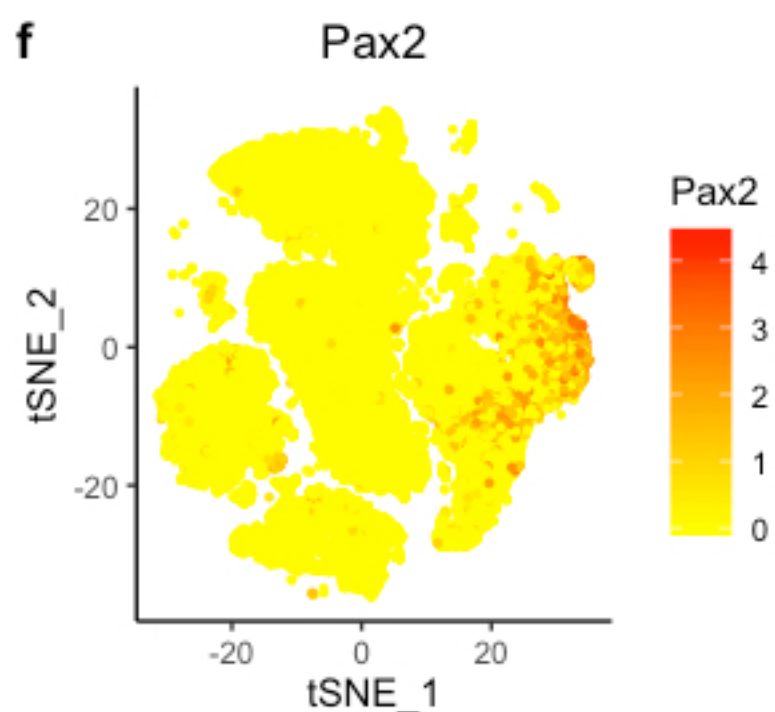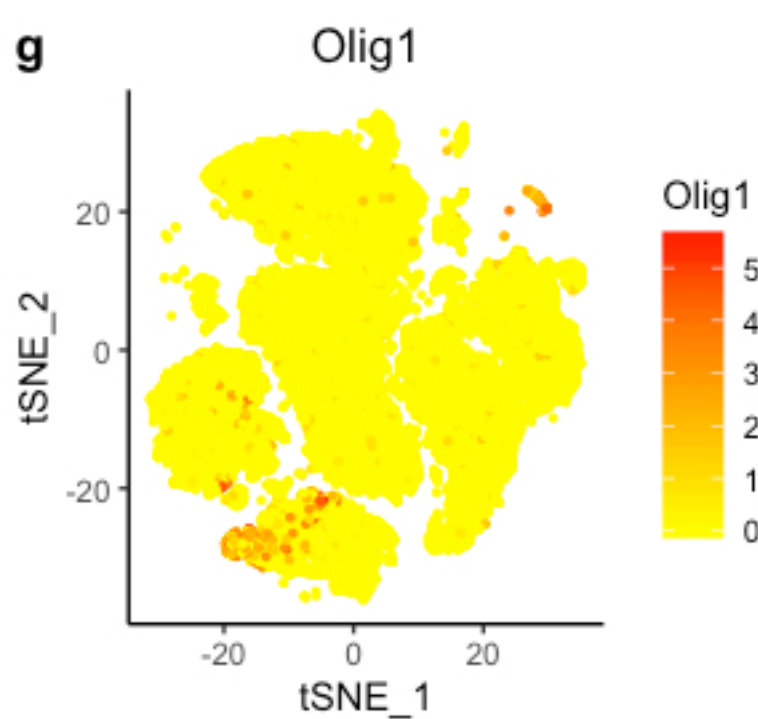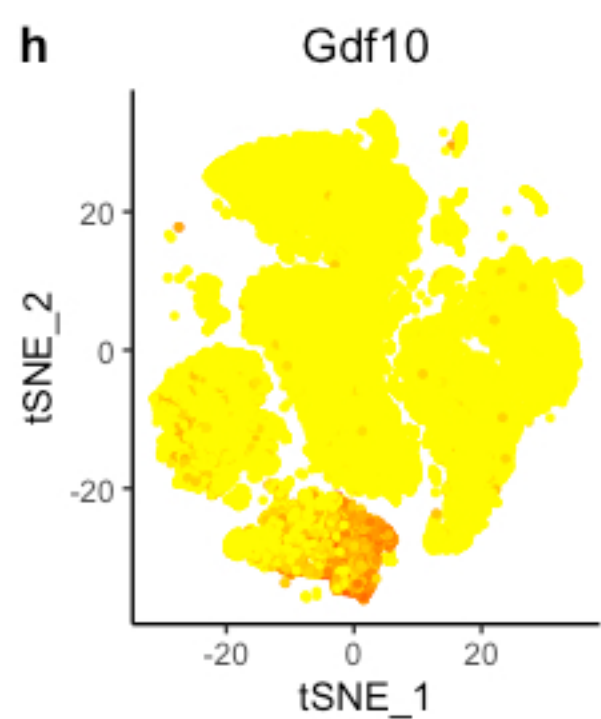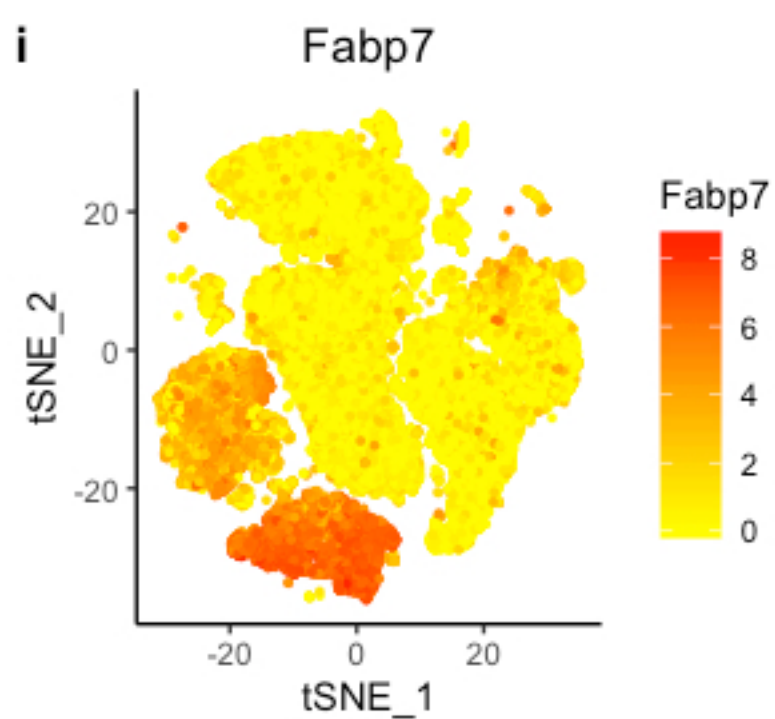

Supplement: S3 Fig — a. tSNE plot showing the ~55,000 neural cells captured in the murine cerebellum data set, colored by cluster number. b-i. Visualization of known marker genes for b. neural stem cells. c. glutamatergic cells, d-f. GABAergic cells, g-i. glias in the murine cerebellum data set. (PDF) [file pcbi.1008205.s003.pdf]

# Ground truth

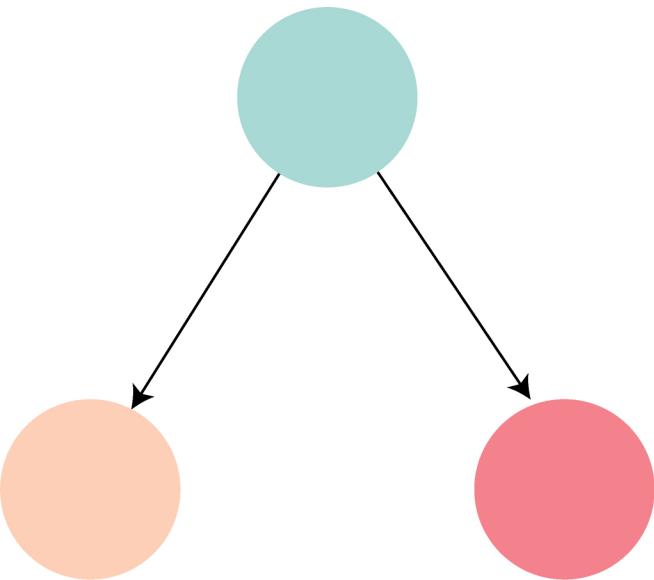

# Overclustering

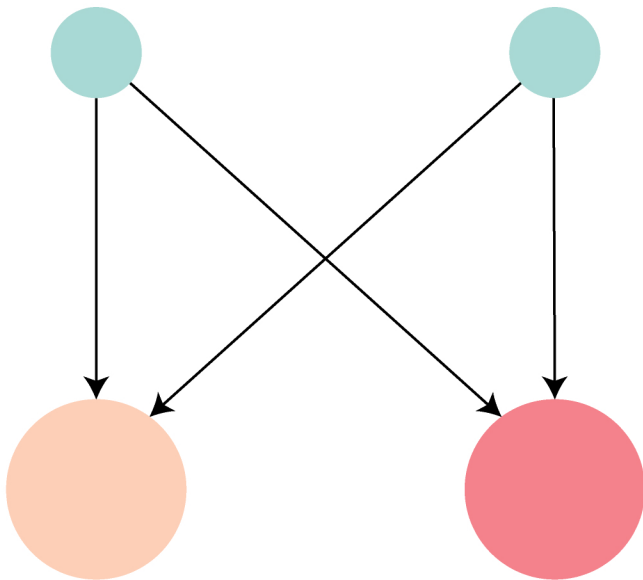

# Underclustering

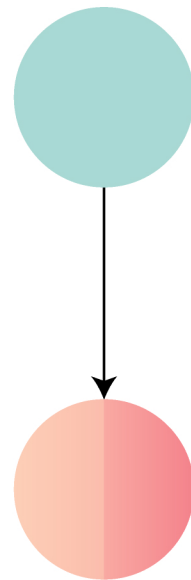

Supplement: S5 Fig — Over clustering (middle) can lead to complex lineages with converging connections, while under clustering (right) can lead to oversimplified lineages. (PDF) [file pcbi.1008205.s005.pdf]

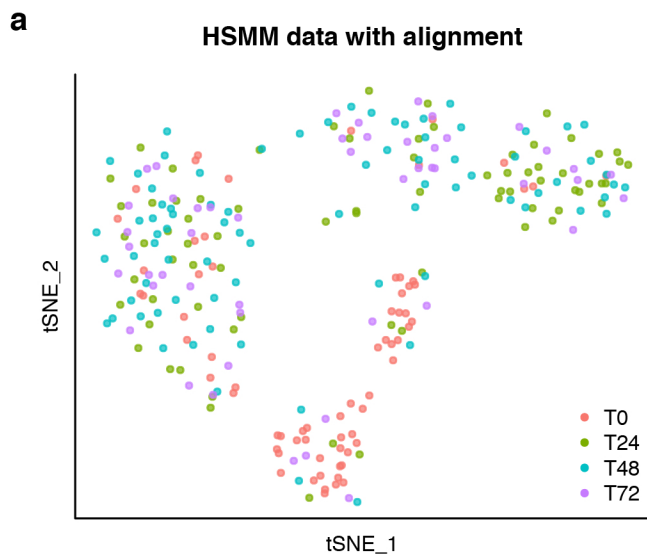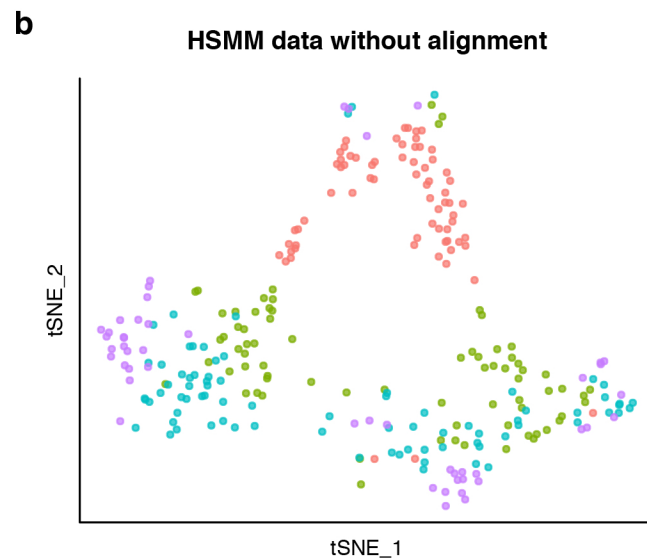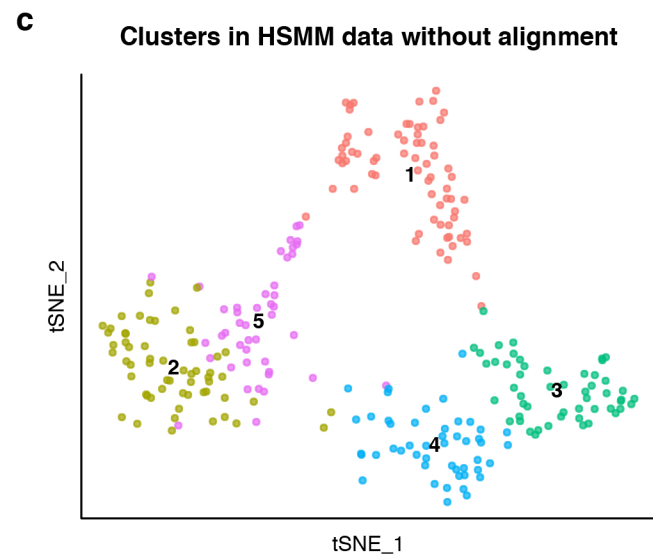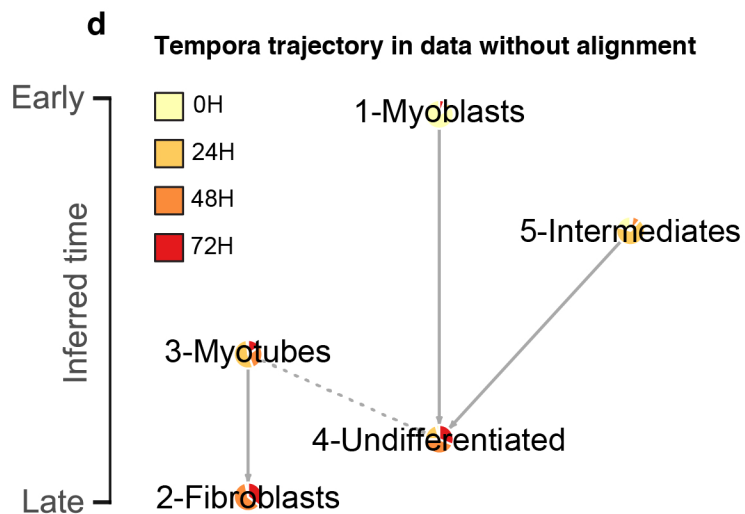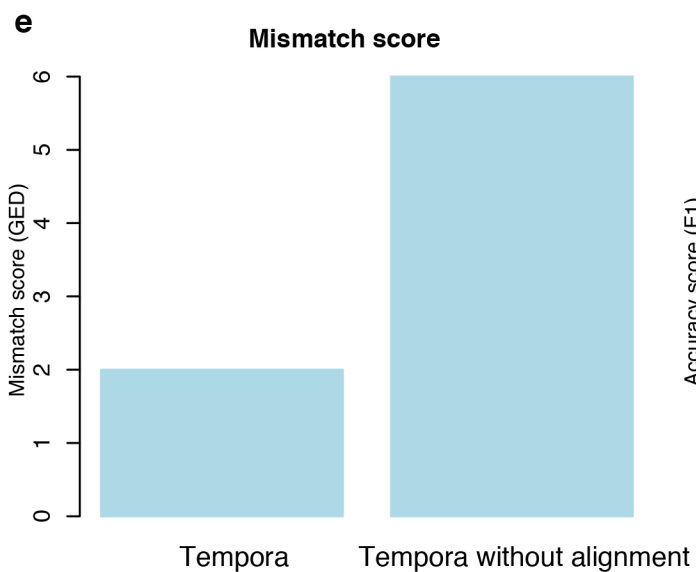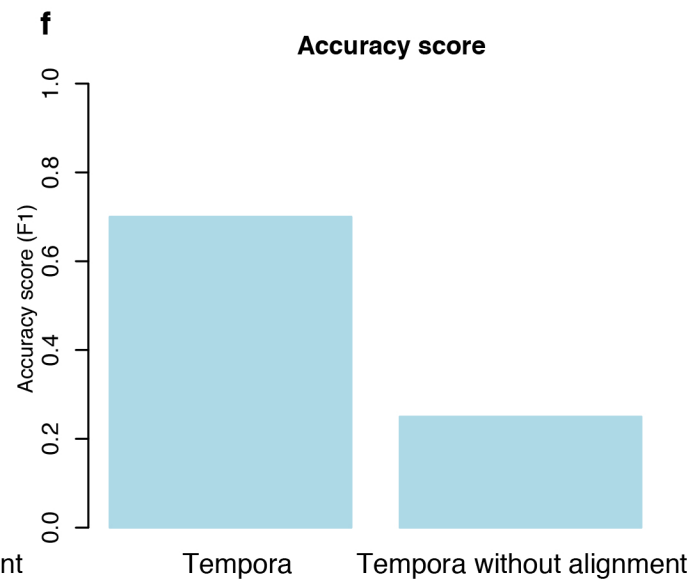

Supplement: S6 Fig — a-b. tSNE plots of HSMM data a. with and b. without Harmony alignment, with cells colored by time points. c. tSNE plot of clusters in HSMM data without alignment. d. Tempora trajectory and e-f. performance evaluation of Tempora on HSMM data without alignment. (PDF) [file pcbi.1008205.s006.pdf]

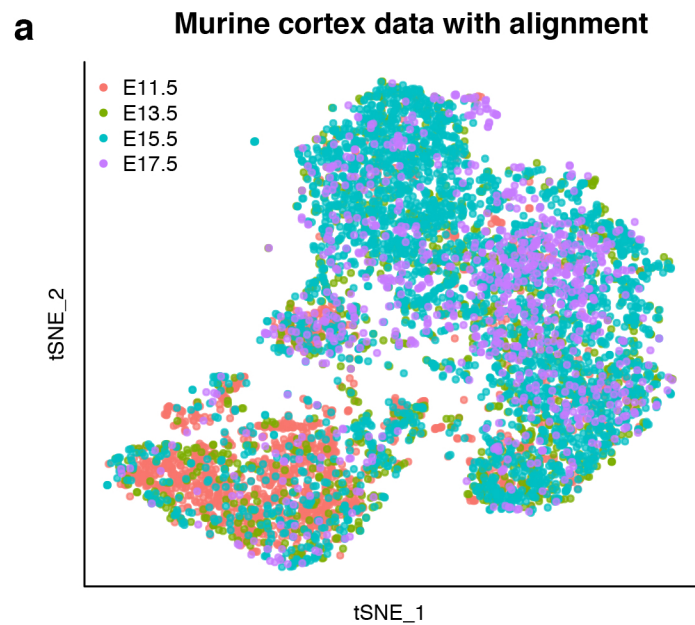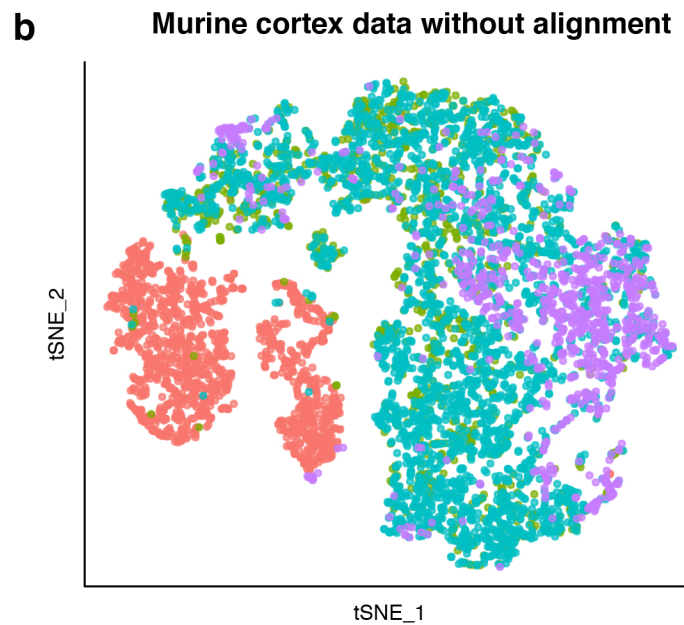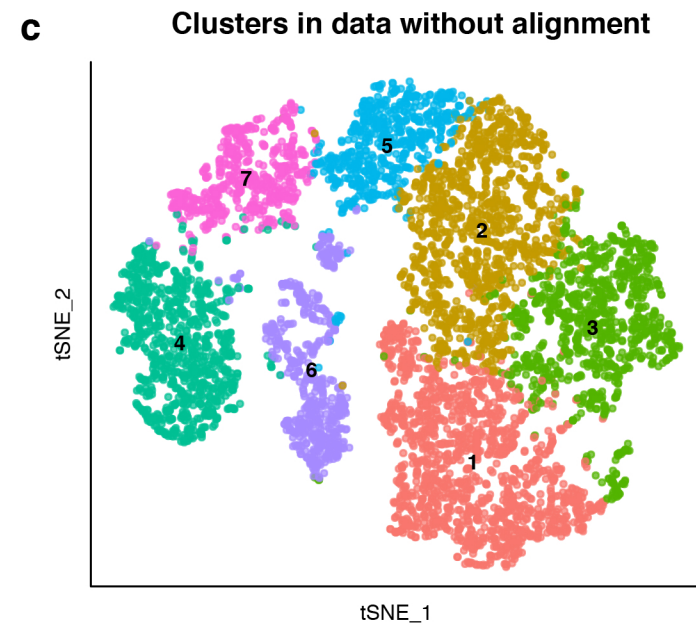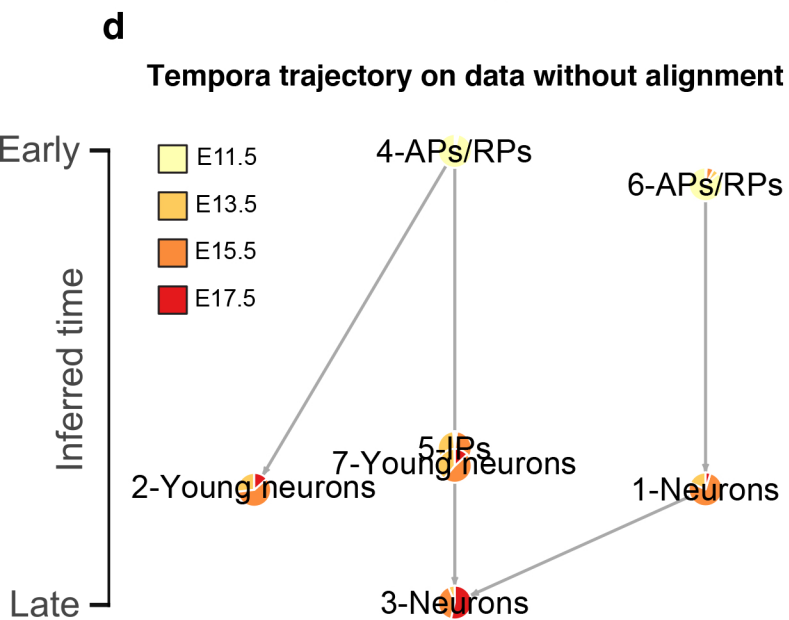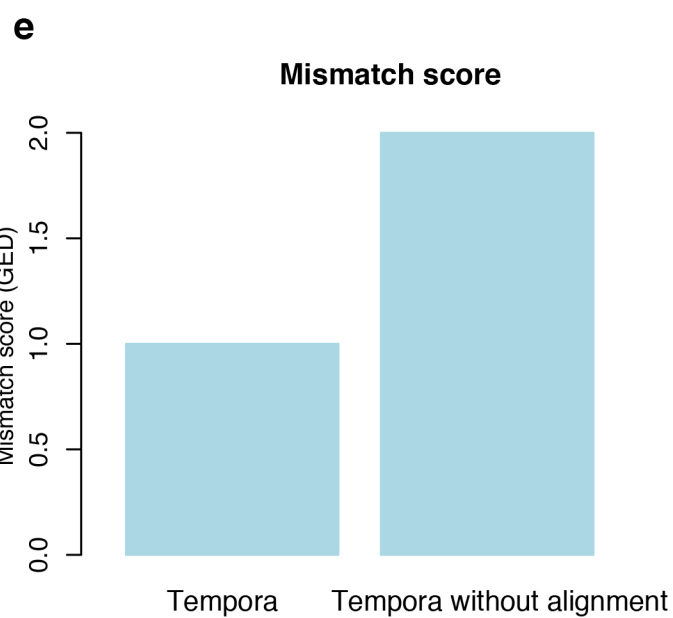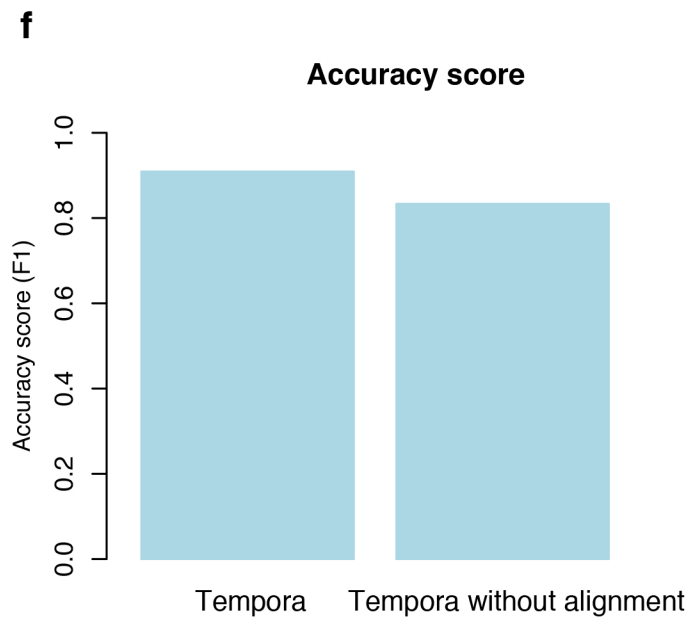

Supplement: S7 Fig — a-b. tSNE plots of murine cerebral cortex data a. with and b. without Harmony alignment, with cells colored by time points. c. tSNE plot of clusters in murine cerebral cortex data without alignment. d. Tempora trajectory and e-f. performance evaluation of Tempora on murine cerebral cortex data without alignment. (PDF) [file pcbi.1008205.s007.pdf]

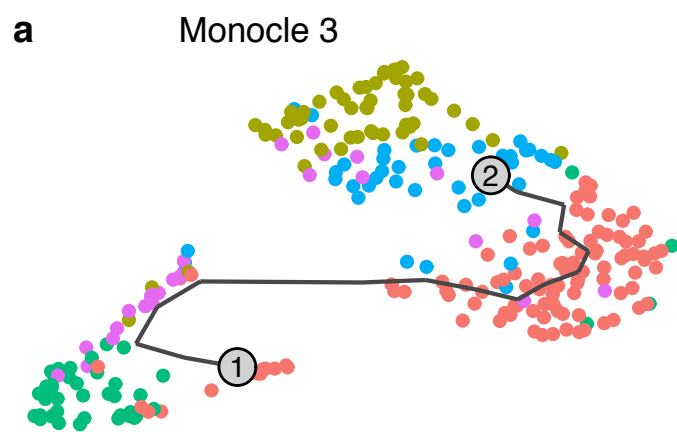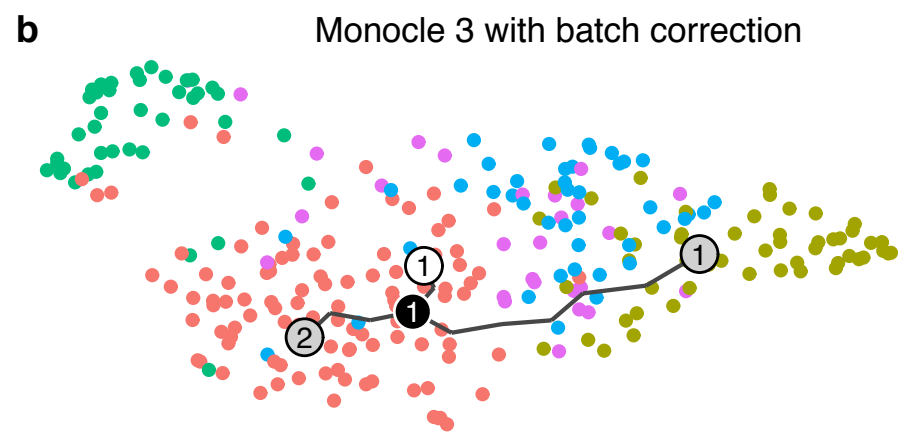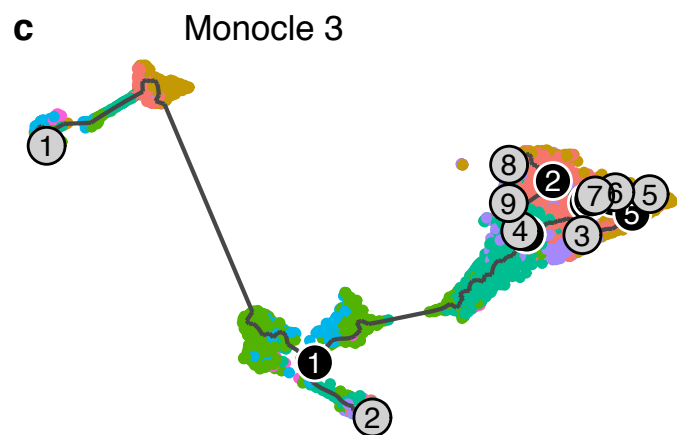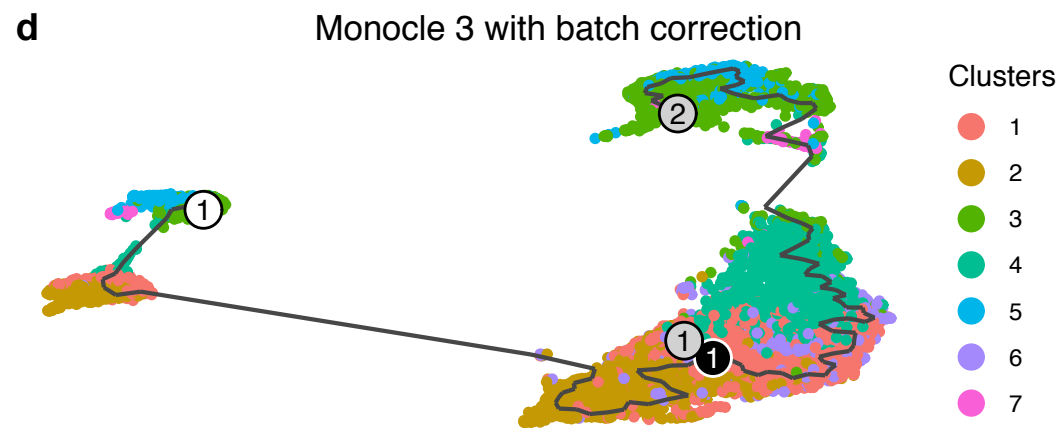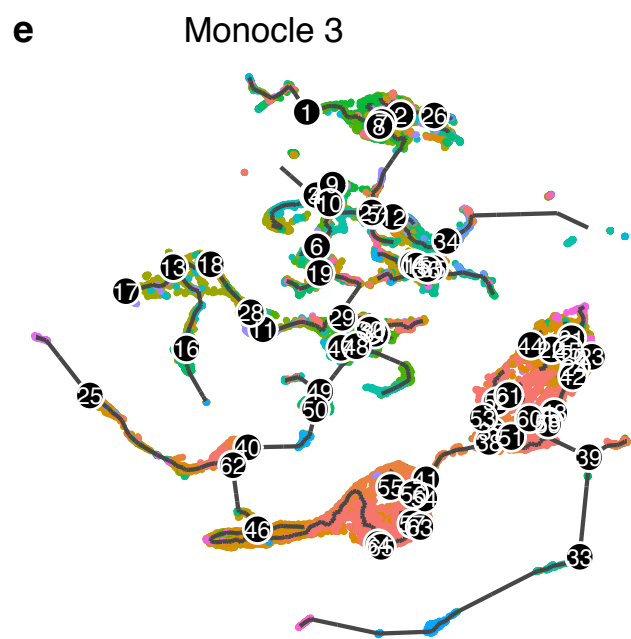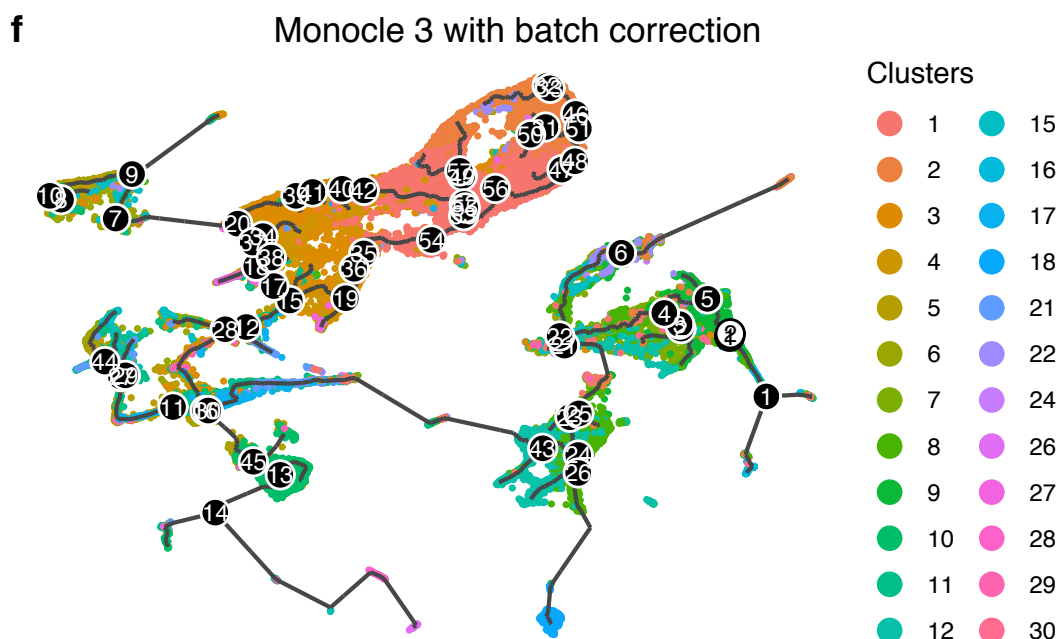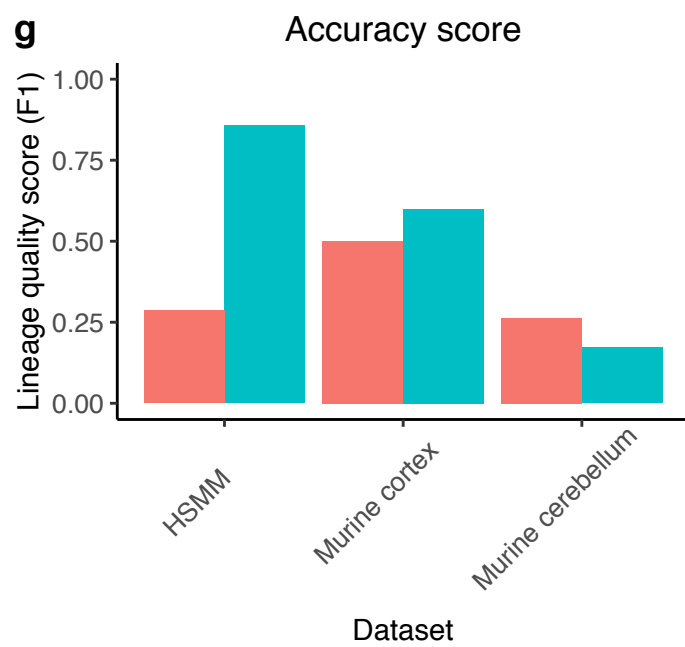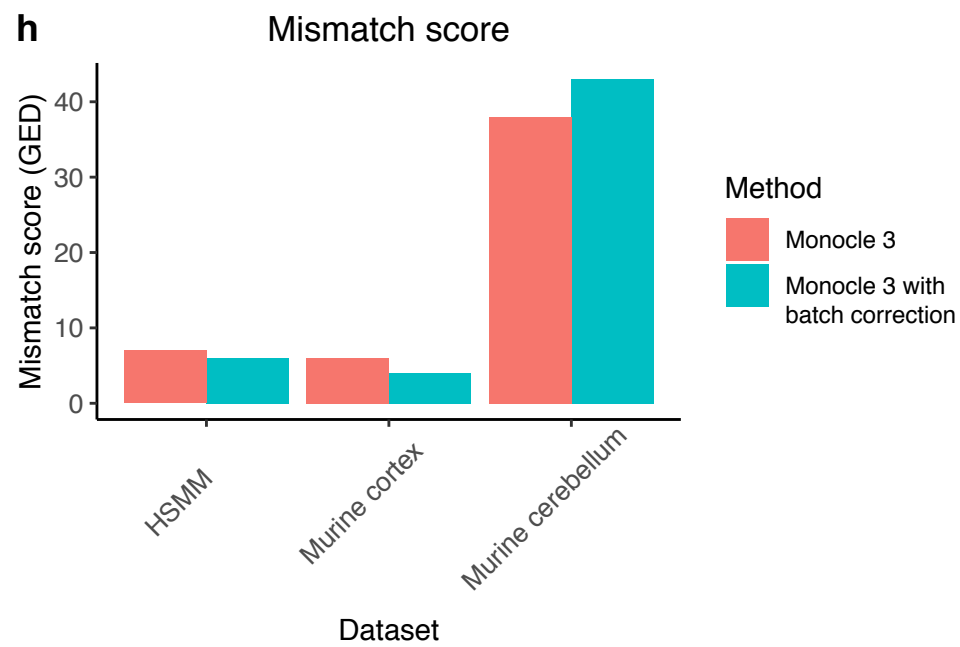

Supplement: S9 Fig — a, c, e. Monocle 3 trajectories of a. HSMM, c. murine cerebral cortex and e. murine cerebellar data sets without batch correction. b, d, f. Monocle 3 trajectories of b. HSMM, d. murine cerebral cortex and f. murine cerebellar data sets with Batchelor batch correction. g-h. Performance evaluation of Monocle 3 on the benchmarking data sets with and without batch effect correction. (PDF) [file pcbi.1008205.s009.pdf]

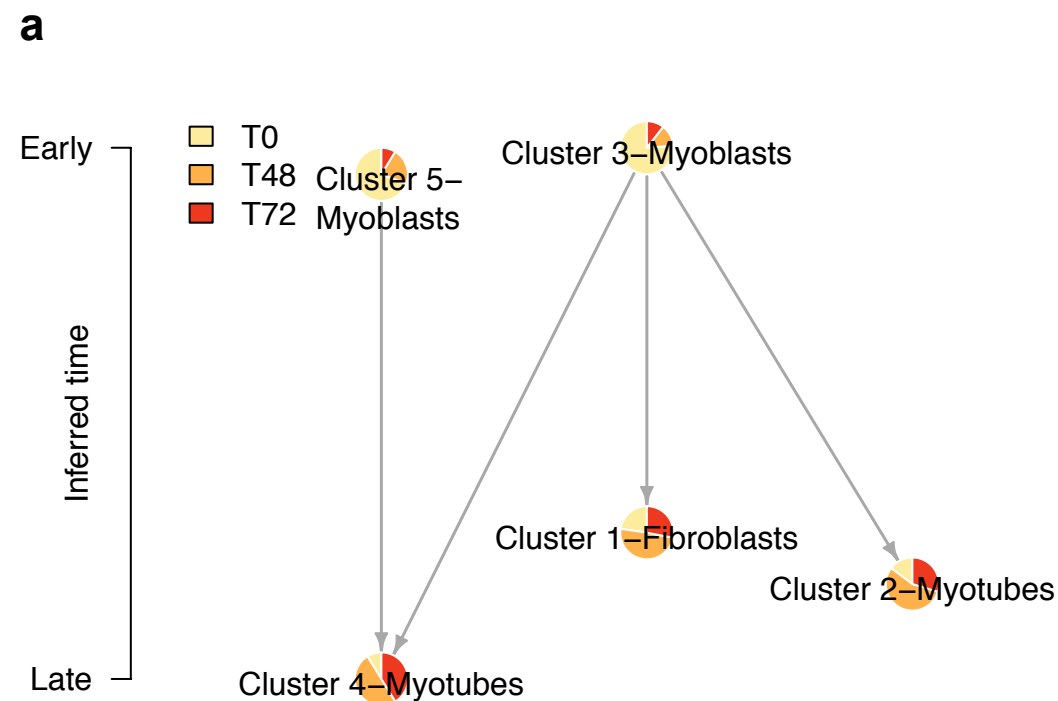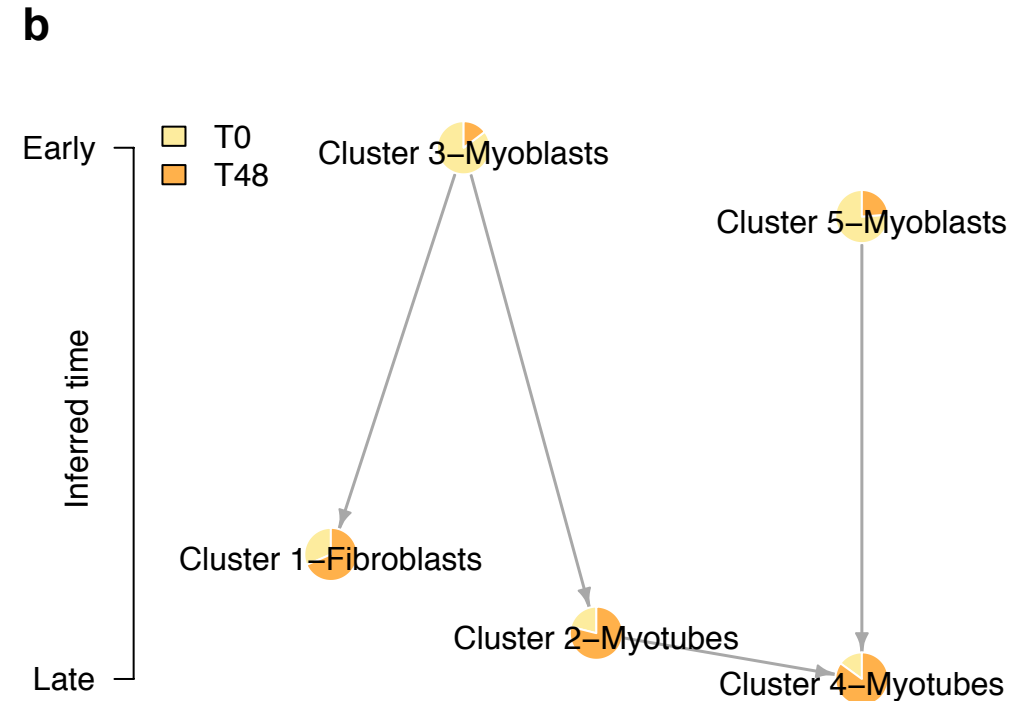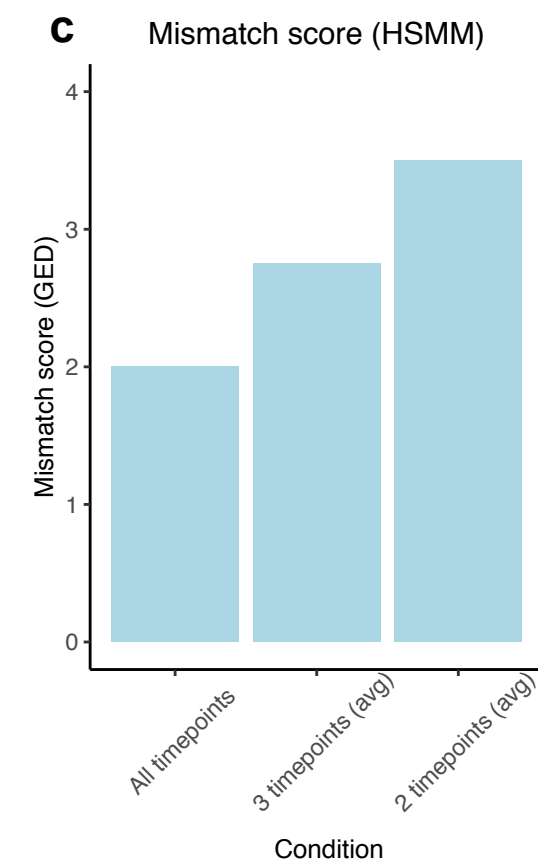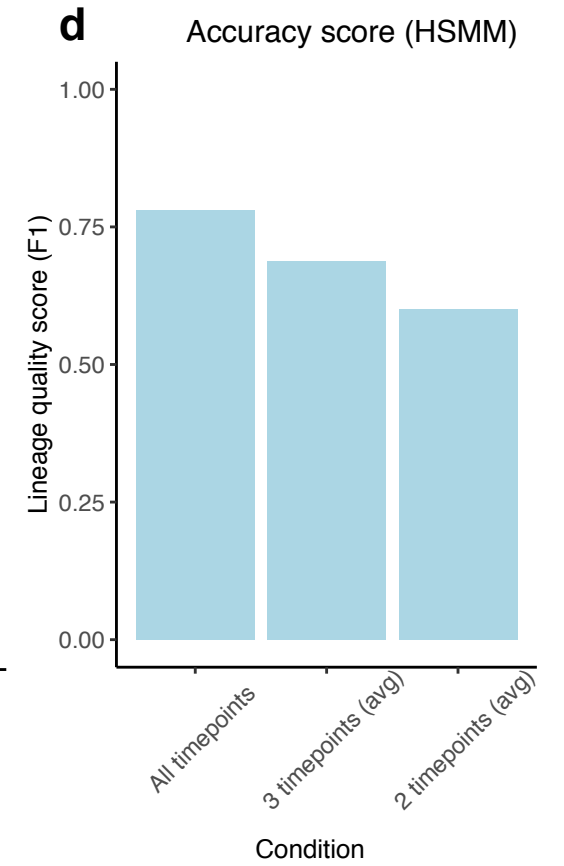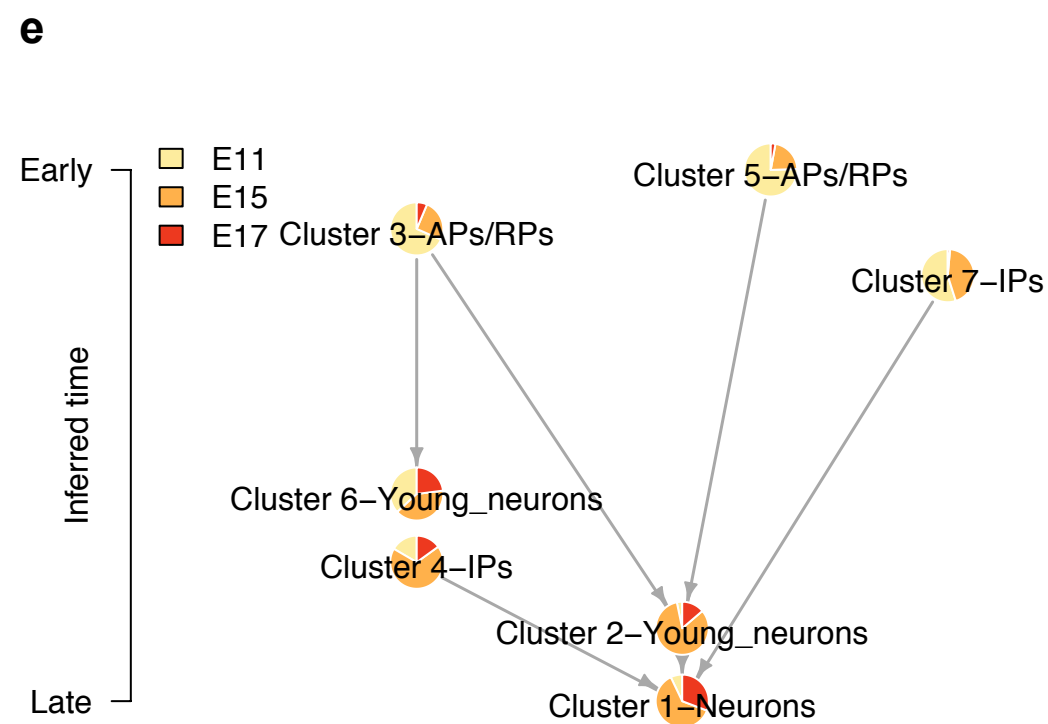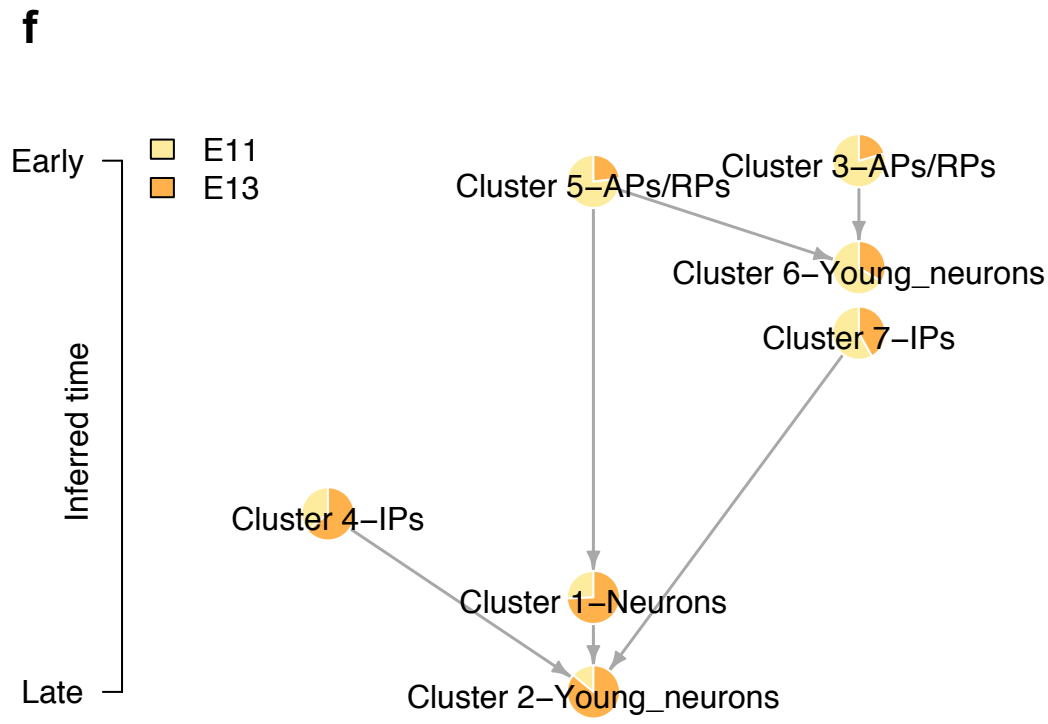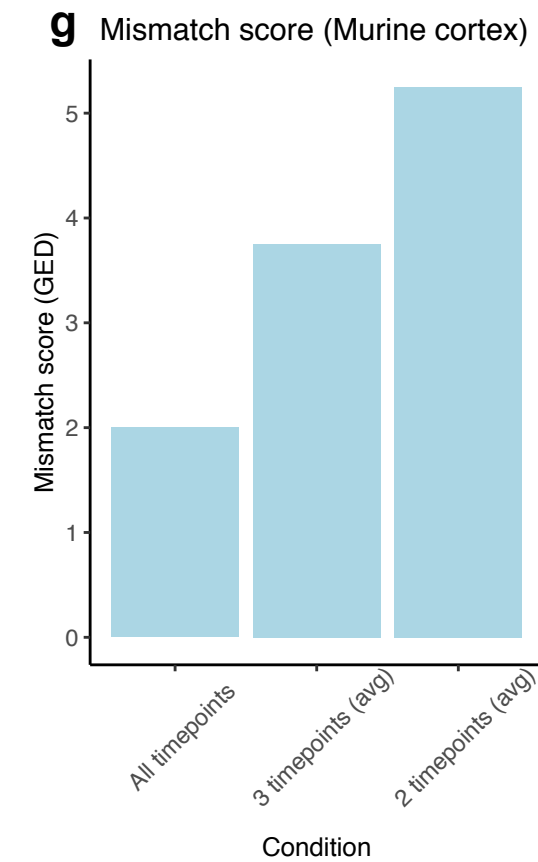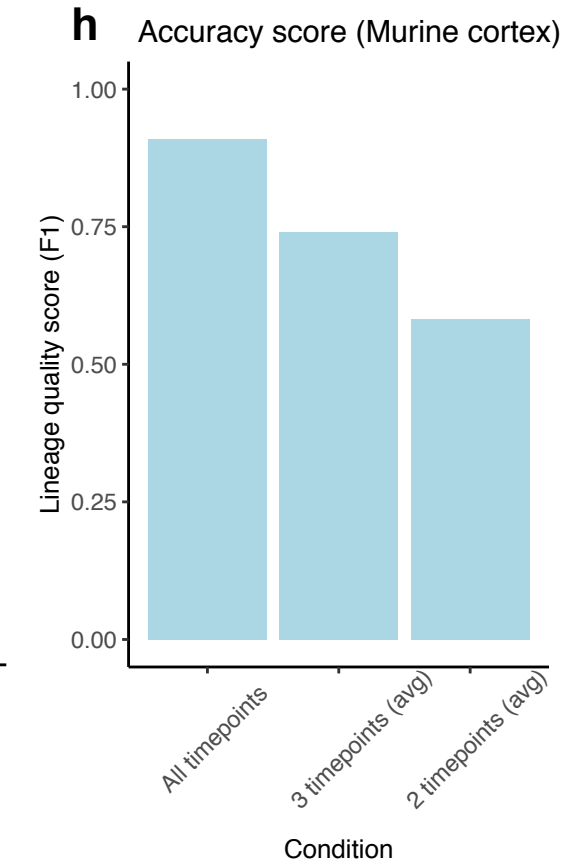

Supplement: S10 Fig — a-b. Tempora trajectory of HSMM data when cells from a. 24 hours and b. 24 hours and 72 hours are removed. c. Mismatch score and d. accuracy score evaluation of Tempora performance on the HSMM data set when time points are down sampled. e-f. Tempora trajectory of murine cerebral cortex data when cells from e. E13 and f. E15 and E17 are removed. g. Mismatch score and h. accuracy score evaluation of Tempora performance on the murine cerebral cortex data set when time points are down sampled. Scores represent an average of four experiments, in which all cells from a different time point or combination of two time points are removed before running Tempora. (PDF) [file pcbi.1008205.s010.pdf]

**a**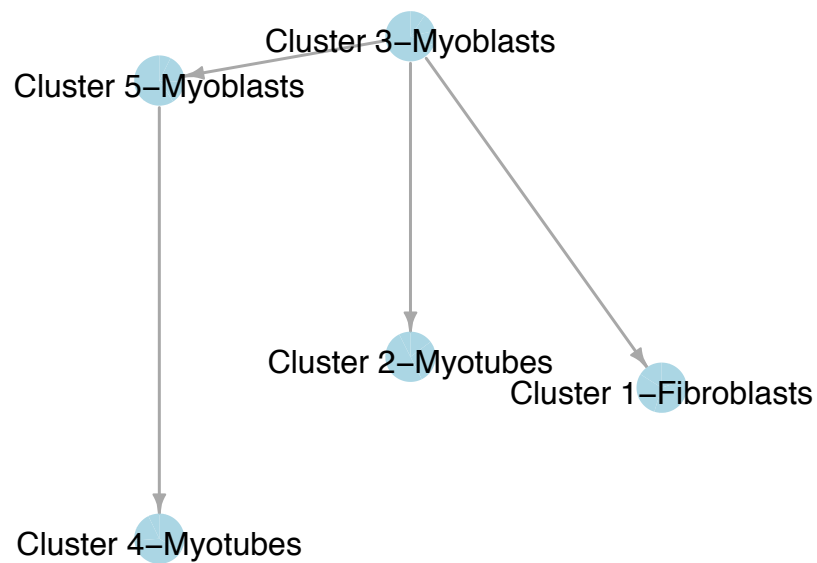**b**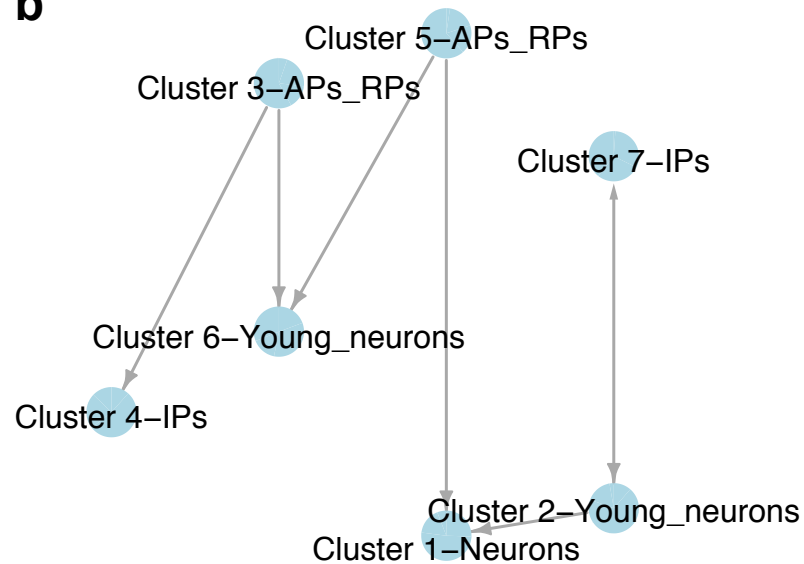**c**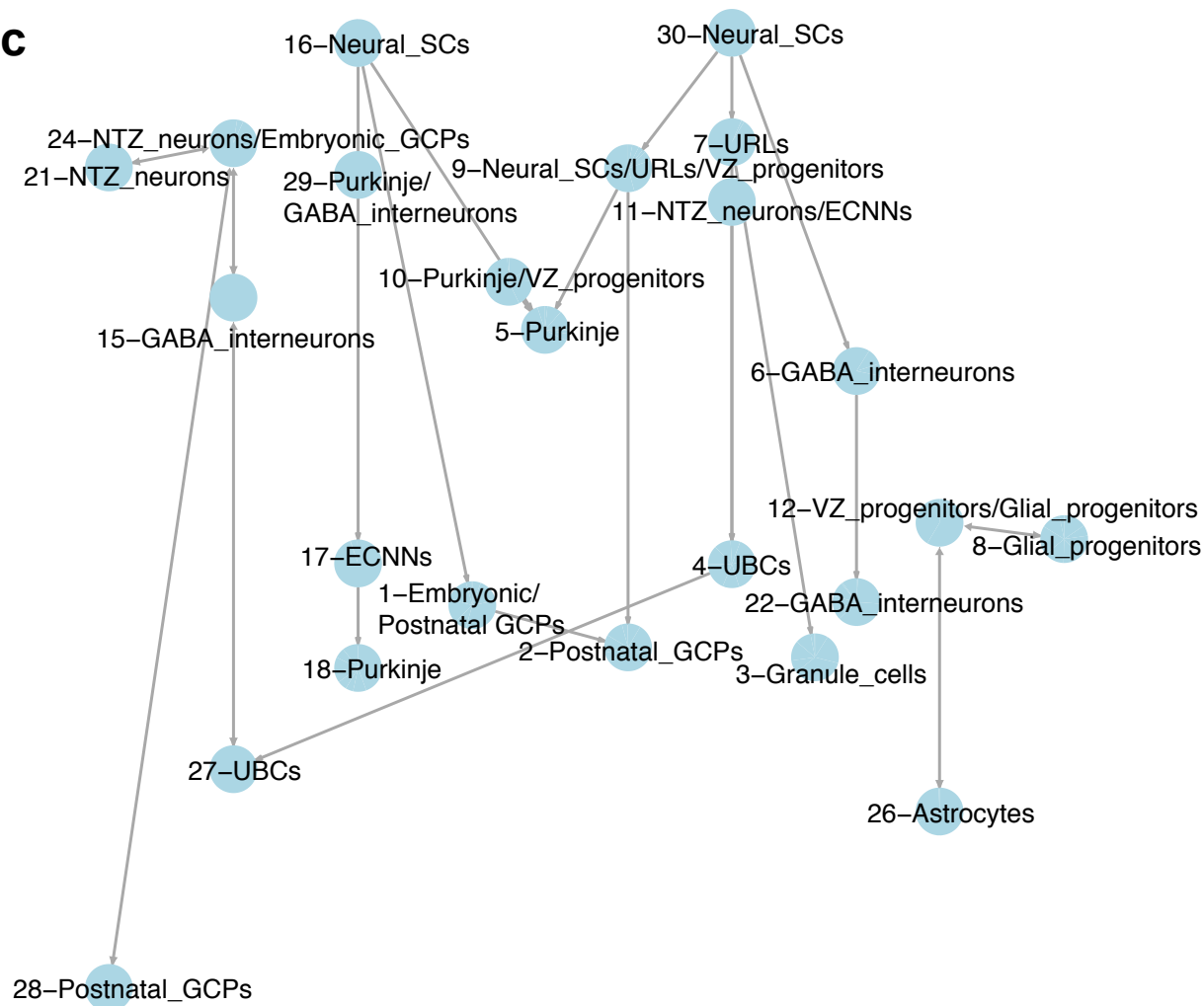**d**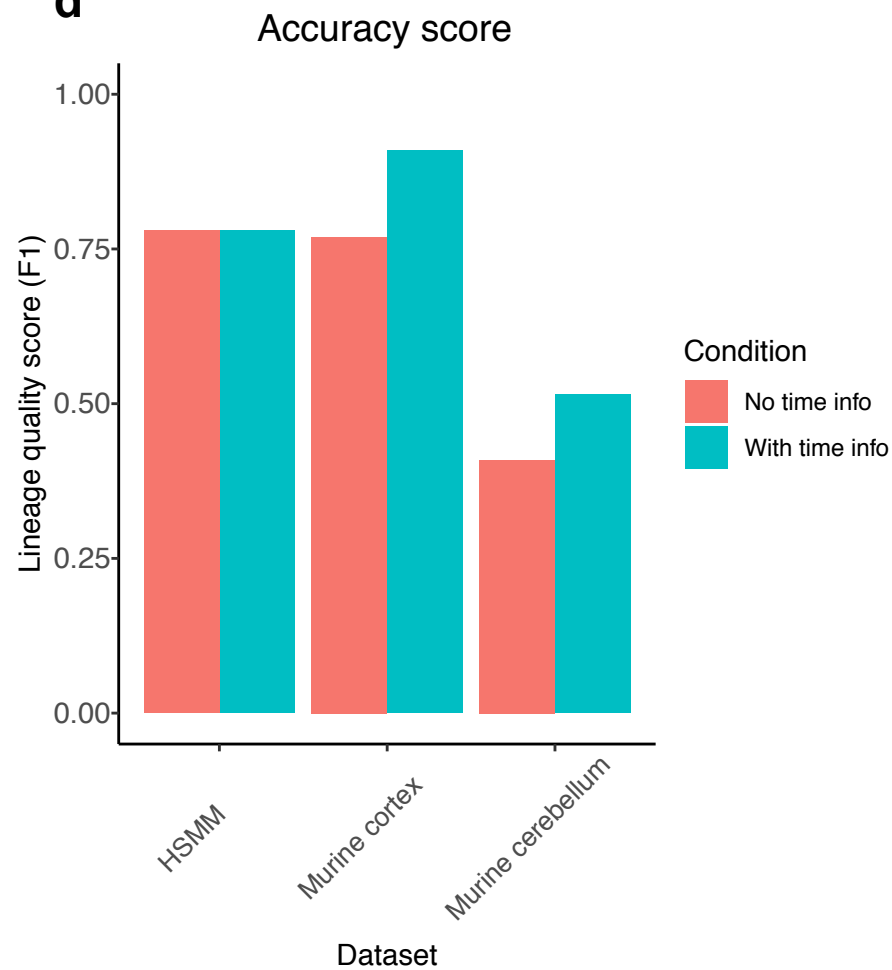

Supplement: S11 Fig — a-c. Tempora trajectories of a. HSMM, b. murine cerebral cortex and c. murine cerebellum data set, with edge directions determined by identifying the root state(s) with known early marker genes (CDK1, CCND5 for myoblasts in the HSMM data set, Sox2 for apical precursors in the murine cerebral cortex data set and Nes for neural stem cells in the murine cerebellar data set) and directing all edges outwards from the root states. d. Accuracy score of Tempora trajectories with edge directions determined without time information. (PDF) [file pcbi.1008205.s011.pdf]
