# Supplementary material for: Tempora: Cell trajectory inference using time-series single-cell RNA sequencing data
Source: PLoS Comput Biol. 2020 Sep 9;16(9):e1008205. doi: 10.1371/journal.pcbi.1008205 (PMC7505465; doi:10.1371/journal.pcbi.1008205)
Supplement: S4 Fig — Correlation plots showing cluster-average gene expression and pathway enrichment profiles in a. HSMM and b. murine cerebral cortex data. (PDF) [file pcbi.1008205.s004.pdf]

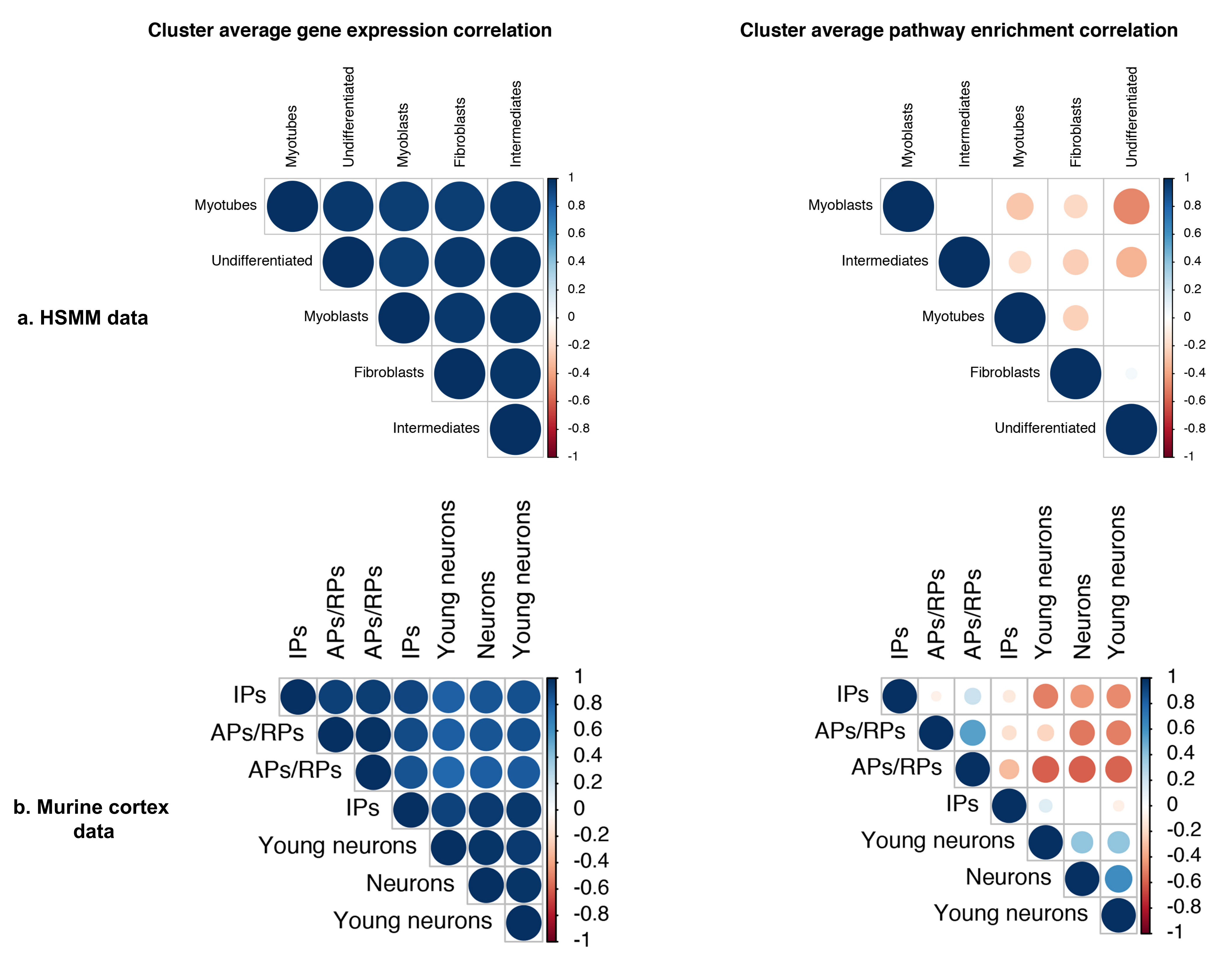
Cluster average gene expression correlation
Cluster average pathway enrichment correlation
a. HSMM data
Myotubes
Undifferentiated
Myoblasts
Fibroblasts
Intermediates
1
0.8
0.6
0.4
0.2
0
-0.2
-0.4
-0.6
-0.8
-1
b. Murine cortex data
IPs
APs/RPs
Young neurons
Neurons
