# Supplementary material for: Tempora: Cell trajectory inference using time-series single-cell RNA sequencing data
Source: PLoS Comput Biol. 2020 Sep 9;16(9):e1008205. doi: 10.1371/journal.pcbi.1008205 (PMC7505465; doi:10.1371/journal.pcbi.1008205)
Supplement: S8 Fig — a-b. tSNE plots of murine cerebellum data a. with and b. without Harmony alignment, with cells colored by time points. c. tSNE plot of clusters in murine cerebral cortex data without alignment. d. Tempora trajectory and e-f. performance evaluation of Tempora on murine cerebellar data without alignment. (PDF) [file pcbi.1008205.s008.pdf]

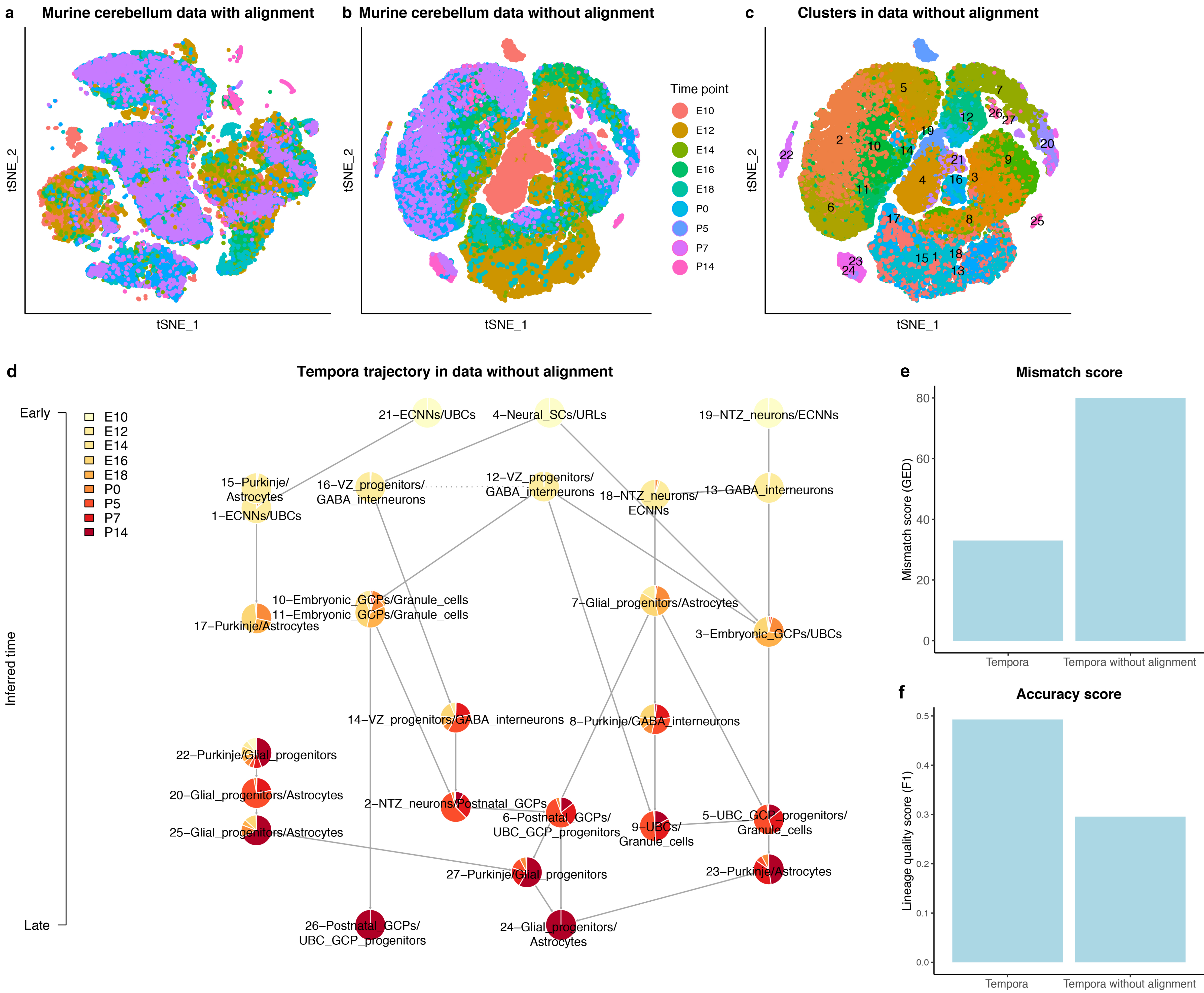

a
Murine cerebellum data with alignment
b
Murine cerebellum data without alignment
c
Clusters in data without alignment
tSNE_1
tSNE_2
Time point
E10
E12
E14
E16
E18
P0
P5
P7
P14
d
Tempora trajectory in data without alignment
Early
Late
Inferred time
21–ECNNs/UBCs
4–Neural_SCs/URLs
19–NTZ_neurons/ECNNs
15–Purkinje/Astrocytes
16–VZ_progenitors/GABA_interneurons
12–VZ_progenitors/GABA_interneurons
18–NTZ_neurons/ECNNs
13–GABA_interneurons
1–ECNNs/UBCs
10–Embryonic_GCPs/Granule_cells
11–Embryonic_GCPs/Granule_cells
17–Purkinje/Astrocytes
7–Glial_progenitors/Astrocytes
3–Embryonic_GCPs/UBCs
14–VZ_progenitors/GABA_interneurons
8–Purkinje/GABA_interneurons
22–Purkinje/Glial_progenitors
20–Glial_progenitors/Astrocytes
2–NTZ_neurons/Postnatal_GCPs
6–Postnatal_GCPs/UBC_GCP_progenitors
9–UBCs/Granule_cells
5–UBC_GCP_progenitors/Granule_cells
25–Glial_progenitors/Astrocytes
27–Purkinje/Glial_progenitors
23–Purkinje/Astrocytes
26–Postnatal_GCPs/UBC_GCP_progenitors
24–Glial_progenitors/Astrocytes
e
Mismatch score
Mismatch score (GED)
0
20
40
60
80
Tempora
Tempora without alignment
f
Accuracy score
Lineage quality score (F1)
0.0
0.1
0.2
0.3
0.4
0.5
Tempora
Tempora without alignment
