# Supplementary material for: Tempora: Cell trajectory inference using time-series single-cell RNA sequencing data
Source: PLoS Comput Biol. 2020 Sep 9;16(9):e1008205. doi: 10.1371/journal.pcbi.1008205 (PMC7505465; doi:10.1371/journal.pcbi.1008205)
Supplement: S12 Fig — Runtime of Tempora when applied to a-b. murine cortex and c-d. murine cerebellum data set after downsampling of a,c. cells and b, d. genes. (PDF) [file pcbi.1008205.s012.pdf]

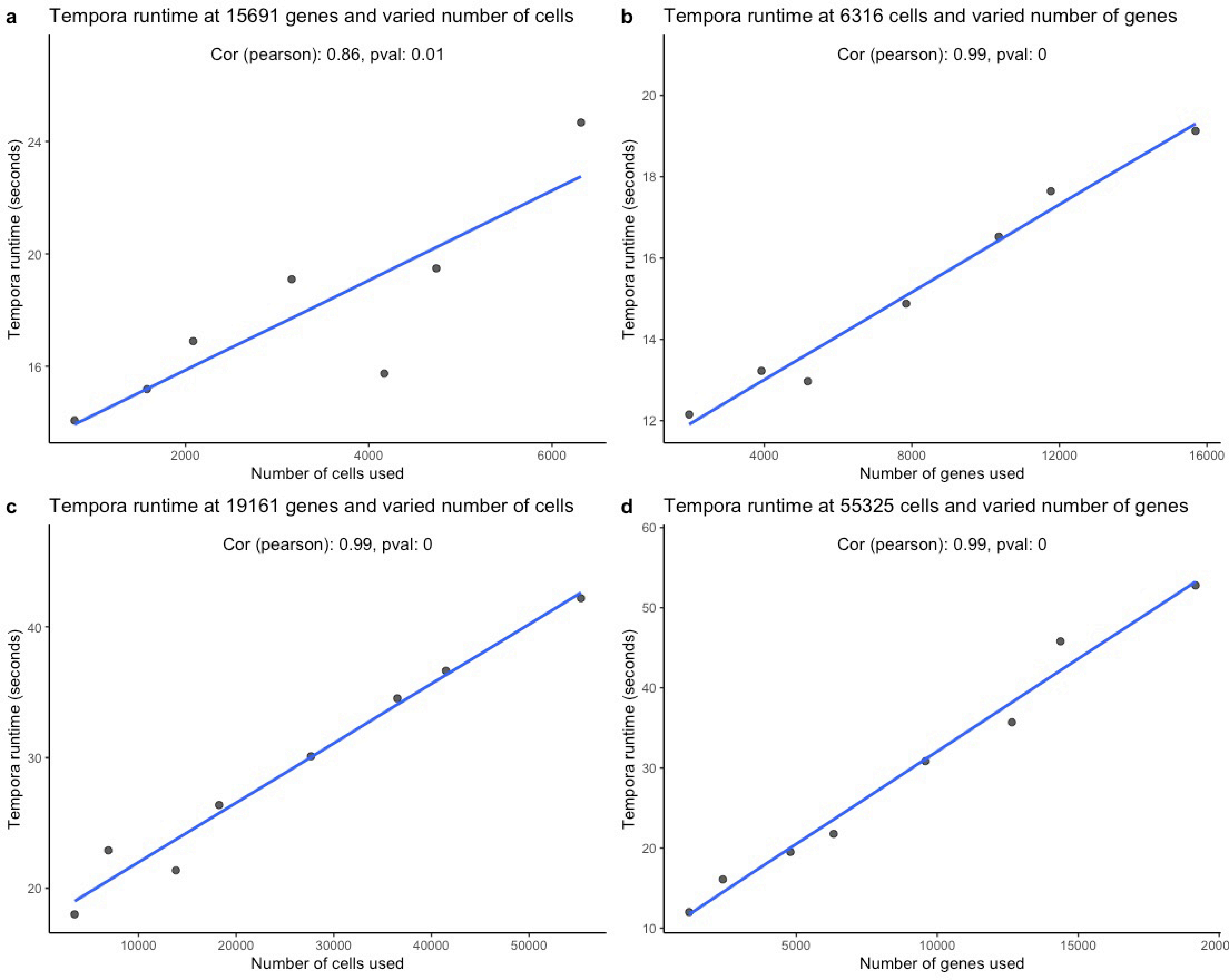

a
Tempora runtime at 15691 genes and varied number of cells
Cor (pearson): 0.86, pval: 0.01
Tempora runtime (seconds)
16
20
24
2000
4000
6000
Number of cells used
b
Tempora runtime at 6316 cells and varied number of genes
Cor (pearson): 0.99, pval: 0
Tempora runtime (seconds)
12
14
16
18
20
4000
8000
12000
16000
Number of genes used
c
Tempora runtime at 19161 genes and varied number of cells
Cor (pearson): 0.99, pval: 0
Tempora runtime (seconds)
20
30
40
10000
20000
30000
40000
50000
Number of cells used
d
Tempora runtime at 55325 cells and varied number of genes
Cor (pearson): 0.99, pval: 0
Tempora runtime (seconds)
10
20
30
40
50
60
5000
10000
15000
2000
Number of genes used
