# Supplementary material for: Tempora: Cell trajectory inference using time-series single-cell RNA sequencing data
Source: PLoS Comput Biol. 2020 Sep 9;16(9):e1008205. doi: 10.1371/journal.pcbi.1008205 (PMC7505465; doi:10.1371/journal.pcbi.1008205)
Supplement: S1 Table — (PDF) [file pcbi.1008205.s013.pdf]

Tempora: cell trajectory inference using time-series single-cell RNA sequencing data

S1 Table. Marker genes used to annotate cell types.

**Data set**	**Cell type**	**Marker genes used**
HSMM (9)	Myoblasts	*CDK1, NCAM*
	Fibroblast	*SPHK1, PDGFRA*
	Myocytes	*MYOG, MYH2, ENO3, MYH3*
	Intermediates	*MEF2C, ID1*
Murine cerebral cortex (5)	Apical precursors	*Sox2, Pax6, Hes1, Mki67*
	Radial precursors	*Edrnb, Vim, Slc1a3*
	IPs	*Eomes, Gadd45g, Mfap4, Sstr2*
	Young neurons	*Tbr1, Tubb3, Foxp2, Reln*
	Neurons	*Tubb3, Bhlhe22, Satb2, Fezf2, Mef2c, Gria2*
Murine cerebellum (34)	Neural stem cells	*Nes, Atoh1, Olig3*
	Nuclear transitory zone (NTZ) neurons	*Atoh1, Lhx2, Lhx9*
	Upper rhombic lip progenitors (URLs)	*Atoh1, Nes, Olig3*
	Excitatory cerebellar nuclei neurons (ECNNs)	*Lmx1a, Lhx2, Lhx9*
	Embryonic granule cell progenitors (GCPs)	*Atoh1, Eomes*
	Postnatal GCPs	*Atoh1, Pdgfra*
	Unipolar brush cells (UBCs)	*Lmx1a, Eomes*
	UBC/GCP progenitors	*Atoh1, Eomes, Pdgfra*
	Granule cells	*Atoh1, Eomes, Pax6*
	Ventricular zone (VZ) progenitors	*Nes, Ptf1a, Ascl1, Fabp7*
	GABA interneurons	*Pax2, Ptf1a, Ascl1*
	Purkinje neurons	*Rora, Calb1*
	Gliogenic progenitors	*Nes, Fabp7, Aldh1l1, Gdf10*
	Astrocytes	*Ptf1a, Fabp7, Aldh1l1, Gdf10, Ascl1, Calb1*
