# Supplementary material for: Tempora: Cell trajectory inference using time-series single-cell RNA sequencing data
Source: PLoS Comput Biol. 2020 Sep 9;16(9):e1008205. doi: 10.1371/journal.pcbi.1008205 (PMC7505465; doi:10.1371/journal.pcbi.1008205)
Supplement: Attachment — Submitted filename: Tempora_Response_to_Reviewers.docx [file pcbi.1008205.s014.docx]

Dear Editor,

Thank you for the opportunity to submit a revised draft of our manuscript titled “Tempora: cell trajectory inference using time-series single-cell RNA sequencing data” to PLOS Computational Biology. We appreciate the time and effort that you and the reviewers have spent providing valuable feedback.

The two common criticisms we received from the reviewers are the small numbers of data sets and limited selection of trajectory inference (TI) methods that were used to validate and benchmark Tempora. To address this, we added additional state-of-the-art TI methods to the comparison with Tempora, including PAGA and Slingshot, two methods that scored the best in benchmarking done by Saelens et al. (Nature Biotechnology 37, 547–554), as well as the most updated version of Monocle and a recent time series-based method, CSHMM. We also included a third dataset of ~55,000 murine neural cerebellar cells collected at nine different time points during brain development (Vladoiu et al., Nature 572, 67–73). Our overall conclusions did not change and Tempora generally outperforms all other methods on all three data sets evaluated here.

We provide a point-by-point response to the reviewers’ comments and suggestions below.

**Reviewer 1:**

Major Comments:

1. It remains unclear how many time series points are necessary for Tempora. Will Tempora be compatible and robust when only two or three time points are available? Are the inferred trajectories for HSMM and murine cortex stable to downsampling of time points?

**Response: We have re-run Tempora on the HSMM and murine cortex data while removing cells from one or two time points to evaluate the effect of time point down sampling on Tempora’s performance. Tempora’s performance, on average, decreases slightly on both datasets when one time point is removed and further decreases when two time points are removed (Supplementary Figure 10). Even though this decrease is observed on average, some time points are less important than others, as removing some time points resulted in similar performance compared to when the full set of time points were used (Fig S10). These results show that Tempora is compatible with data containing as few as two time points, however, including additional time points is beneficial.**

2. A number of unified single-cell analysis approaches have recently been published to accommodate joint embeddings across batches of data such as time series data, including Harmony (Korsunsky et al, Nature Methods 2019) and BBKNN (Polanski et al Bioinformatics 2019) to name a few. Indeed, the authors use Harmony corrected PCs prior to Tempora to ensure that trajectories are not driven by batch-effects. It is currently unclear from the Methods section whether similar batch-correction treatments were applied in the Monocle and TSCAN comparison, such that inferior performance noted for Monocle and TSCAN could be attributed to the lack of controlling for batch-effects.

**Response: Monocle’s latest version (v3) now provides an align_cds() function, which optionally aligns data sets from different time points using Batchelor (Haghverdi et al., Nature Biotechnology 2018). This enables us to compare Tempora to Monocle with and without batch correction and using the author’s recommended workflow. Batch correction dramatically improved Monocle 3 performance on the muscle data (HSMM), but only slightly on the cerebral cortex data, and reduced performance on the murine cerebellar data (Figure S9). For methods we applied through the Dynverse wrapper, including PAGA, Slingshot and TSCAN, since their pipelines include preprocessing of data and the downstream trajectory inference depends upon certain preprocessing steps, we avoided applying the same batch effect correction to these methods. CSHMM requires a gene expression matrix as input, which is incompatible with Harmony’s output of principal components. For selected methods we compare with Tempora, we followed recommended workflows where available as this is closer to how a normal user would experience these packages. We suggest that future work could assess the effects of various batch correction methods on various trajectory inference methods.**

3. A major limitation of Tempora is that cells are collapsed into populations and the populations are ordered in a trajectory, rather than the cells themselves, thereby losing the single-cell resolution of inferred pseudotemporal ordering. It is unclear why Tempora elects to collapse cells into populations given the dynamic and continuous nature of developmental processes. A discussion of the benefits and limitations of this approach is warranted.

**Response: While scRNA-seq offers the opportunity to identify the transitions cells undergo during a dynamic process at cellular resolution, the granularity of a trajectory inferred at the single-cell level can often render it difficult to interpret, especially for large data sets. Furthermore, current scRNA-seq technologies (e.g. 10x) typically have low gene expression sensitivity at the single cell level, which is a challenge for analysis methods. By analyzing scRNA-seq data at the cluster level instead of at the single-cell level, Tempora: 1) amplifies gene expression signals from one cell using similar cells in a cluster; 2) eases interpretation, because it operates on a relatively small number of clusters which usually represent known cell types, and which naturally fits with the standard practice of clustering scRNA-seq data to identify cell types; and 3) reduces computational time and resources needed to analyze large data sets because it works with a relatively small number of clusters instead of a potentially very large number of cells. However, a disadvantage of Tempora’s cluster-based approach is that it does not identify gene expression gradients over cells observable in scRNA-seq data. We propose that Tempora’s approach is complementary to trajectory inference methods that work at the single cell level and could be used together with these other methods to analyze both cell clusters and single cells. These points have been added to the manuscript.**

**The Tempora framework could be extended in the future to study specific transitions from the constructed trajectory at the single-cell level, which would enable users to leverage the power of single cell measurements in understanding dynamic processes while still preserving the interpretability of the cluster-based trajectory.**

4. While the authors note over 70 available trajectory inference approaches have been developed, only two (Monocle and TSCAN) are directly compared to Tempora. While it is beyond the scope of this paper to compare Tempora to all published trajectory inference methods, an explanation of why these two particular methods were chosen is warranted.

**Response: The original manuscript compared Tempora to Monocle 2, which has been among the most commonly-used trajectory inference methods, as well as TSCAN, a cluster-based trajectory inference method similar in principle to Tempora (in that it uses clusters), but without using time information. We agree that adding other methods is a good idea. We selected two additional trajectory inference methods that score highly in Dynverse’s evaluation (Saelens et al., Nature Biotechnology 37, 547–554) - PAGA, Slingshot - and updated Monocle to the latest version (v3) (Figures 5-7). We also include CSHMM, which is a recent method that considers time point ordering information, like Tempora. We also include more explanation in the manuscript about why these five methods were selected.**

5.  Only two time-series datasets were evaluated here (HSMM and murine cortex), though more are available, including Zebrafish (Farrell et al, Science 2018) and human preimplantation embryos (Petropoulos, Cell 2016) to name a few. Notably, the two time-series datasets evaluated by Temporal currently have 271 and 6,000 cells respectively while approximately 40,000 cells are available in the Zebrafish dataset, spanning many more distinct lineages and complex trajectories. Particularly as larger and larger scRNA-seq datasets are generated, it will be important to assess Tempora on a large dataset to better evaluate its scalability, usability, and accuracy for more complex trajectories.

**Response: We agree with this comment and appreciate the suggestions of available data sets. We have additionally evaluated Tempora on a data set of ~55,000 single cells from nine time points throughout the murine neural cerebellar development process (Figures 4, 7). We chose this data set because it contains a relatively large number of cells and the lineages it contains, though complex, are well-studied and the original publication included a hand curated ground-truth trajectory map that we could use to evaluate our prediction.**

---

Minor comments:

1. The authors note that Tempora begins with an optimized clustering of scRNA-seq data and that over or under clustering may result in inaccurate trajectories. Readers and users would greatly benefit from some general guidelines to help them determine whether their data are over or under clustered. The current level of description in the Methods and Discussion requires further elaboration.

**Response: We have included further discussion on this point in the manuscript.**

2. The authors note that Tempora assumes that cell differentiation progresses unidirectionally from stem/progenitor states to differentiated states. It seems that application to aberrant disease programs such as cancer where these assumptions are violated would produce erroneous results. A discussion of this caveat should be noted in the discussion to mitigate user error.

**Response: Thanks for highlighting this. We have added this point to the manuscript. Expanding Tempora to infer trajectories in applications that would include dedifferentiation is an interesting direction for future work.**

**3.** Without lineage tracing data, it is unclear how the current gold-standard was established. Is it resolution of the gold-standard at the population level or single-cell resolution? Please clarify.

**Response: All gold standard trajectories are hand annotated from the literature and based on cell population level information. We manually curated the gold standard trajectories for the human myoblast and murine cortex data sets from the literature, identifying papers based on standard cell and developmental biology experiments. We identified these papers by searching PubMed using the cell types present in our data set as keywords, with no restriction to time, and selected review articles describing relationships between these cell types as well as original research articles with experiments demonstrating relationships or transitions between the cell types of interest. The full list of publications used for the annotation of the human myoblast and murine cerebral cortex gold standard trajectories is cited in the main text. The gold standard trajectory for the murine cerebellum data set is taken from the original publication, where it was hand annotated by the authors who are experts in cerebellar development.**

4. The level of detail in terms of parameters used for Harmony, Monocle, TSCAN currently presented in the Methods section needs further elaboration to ensure reproducibility.

**Response: We have better specified the parameters used in each trajectory inference method in the Methods section.**

**Reviewer #2:**

Major points:

**(1)** There are already a number of computational methods developed for time-series scRNA-seq data. For example, TASIC (Bioinformatics 2017: <https://doi.org/10.1093/bioinformatics/btx173>), SINCERITIES (Bioinformatics 2018: <https://doi.org/10.1093/bioinformatics/btx575>), CSHMM (Bioinformatics 2019: <https://doi.org/10.1093/bioinformatics/btz296>), TSEE (BMC Genomics 2019: <https://doi.org/10.1186/s12864-019-5477-8>),Waddington-OT (Cell 2019: <https://doi.org/10.1016/j.cell.2019.01.006>). However, the authors did not cite and compare with any of them.

**Response: As Tempora outputs a directed acyclic graph (DAG) representation of a trajectory (like most trajectory inference methods), we originally limited our comparison to methods with comparable output. The Waddington-OT method outputs a large network of individual cells connected by ‘temporal coupling’ links which it computes using an optimization method. We could not compare to it because it does not output a DAG, however, we now discuss it. Both TASIC and CSHMM output a DAG and are comparable to Tempora. Both methods are based on a hidden markov model approach. We added a new comparison of Tempora to CSHMM as it is the more recent method of the two and has been shown to be more compatible with larger data sets, while TASIC’s utility is restricted to data sets with a small number of time points, as described in the original publication. SINCERITIES and TSEE are designed for analyzing gene regulatory networks and gene expression patterns in time-series scRNA-seq data, respectively, so their aims and outputs are not comparable to Tempora.**

**We have extended the discussion of time-series scRNA-seq analysis methods in the introduction and discussion based on this comment.**

**(2)** The novelty and strength of Tempora are not clear state. In order to inference cell cluster networks by the existing method ARACNE, Tempora has to treat single-cell data as bulk sequencing data by taking the averaged gene expression profiles, or centroids, of all clusters. A lot of information, such as the cell heterogeneity information (stochasticity of cells) offered by single-cell sequencing are not modeled and lost.

**Response: As mentioned in response to reviewer 1, one of Tempora’s main features is the construction of trajectory at the cluster level, instead of at the single cell level. While scRNA-seq offers the opportunity to identify the transitions cells undergo during a dynamic process at cellular resolution, the granularity of a trajectory inferred at the single-cell level can often render it difficult to interpret, especially for larger data sets. Furthermore, current scRNA-seq technologies (e.g. 10x) typically have low gene expression sensitivity at the single cell level, which is a challenge for analysis methods. By analyzing scRNA-seq data at the cluster level instead of at the single-cell level, Tempora: 1) amplifies gene expression signals from one cell using similar cells in a cluster; 2) eases interpretation, because it operates on a relatively small number of clusters which usually represent known cell types and which naturally fits with the standard practice of clustering scRNA-seq data to identify cell types; and 3) reduces computational time and resources needed to analyze large data sets because it works with a relatively small number of clusters instead of potentially a very large number of cells. We also introduce a novel use of biological pathway prior knowledge, which we show improves trajectory inference accuracy. We have included discussion of these points in the revised manuscript.**

**The Tempora framework could be extended in the future to study specific transitions from the constructed trajectory at the single-cell level, which would enable users to leverage the power of single cell measurements in understanding dynamic processes while still preserving the interpretability of the cluster-based trajectory.**

(3) Please clear state why pathway information can help to identify cell cluster/type transition processes. The pathway information can be incomplete for the biological processes studied, will the method still work? The single cell data have a high rate of drop out event. Will the results for the pathway enrichment analysis be sensitive to drop out event?

**Response: We examined the impact of using pathway information in inferring trajectories among cell clusters/types by applying Tempora with and without the pathway enrichment step. Results in Figure 8 show that without pathway enrichment analysis, the inferred trajectories suffer from substantially worse performance (lower accuracy and higher mismatch scores). Investigating the source of this difference, we found that Pearson correlation coefficients computed between the gene expression profiles of clusters (cell types) were excessively noisy, strongly linking all clusters to each other with poor differentiation ability, which is likely the main cause of the poor performance of other methods. On the other hand, Pearson correlation coefficients of pathway enrichment profiles of all pairs of clusters in the human myoblast and mouse cortex data sets are negative for clusters of different cell types (neurons vs. APs, myoblasts vs. fibroblasts) and positive for clusters of similar cell types (neurons vs. neurons) (Supplementary Figure 4). These results suggest that pathway enrichment information is useful inferring transitions between clusters/states because it better identifies similarities and differences in cell clusters. It is known that pathway enrichment of gene expression profiles increases sensitivity and statistical power, as well as reduces noise (e.g. https://www.nature.com/articles/s41596-018-0103-9 and https://pubmed.ncbi.nlm.nih.gov/26125594/). We interpret our results to indicate that these advantageous properties of pathway information are the reason why using it performs better than using gene expression profiles at correctly identifying cell cluster/type transitions. This is now mentioned in the discussion.**

**While pathway information is certainly incomplete, we have collected data from multiple sources to increase the coverage of genes to a relatively high level. The pathway information that Tempora uses is all manually curated from leading pathway databases, including Reactome, HumanCyc, KEGG, NetPath, Panther, NCI PID and Gene Ontology Biological Process, not including annotations collected electronically (IEA evidence code). The full details of the pathway databases included are provided at http://baderlab.org/GeneSets which is cited in the manuscript. It is true that if there is low pathway coverage of a particular system under study that this could negatively affect the results of Tempora analysis. We have now mentioned this in the discussion.**

**It is true that single cell data tends to suffer from a high dropout rate and low sensitivity. This is one of the main reasons we have chosen to amplify the gene expression signals by averaging gene expression profiles of many cells in each cluster before applying downstream pathway enrichment analysis. It is possible that some bias exists in the data that correlates with pathway information - in this case, we may observe pathway enrichment analysis to have reduced sensitivity. We now include a mention of this caveat in the discussion.**

(4) How many parameters does Tempora have? How sensitive are they to the results? For example, in the implementation of ARACNE, is there any score for removing the edges?

**Response: Tempora has three parameters, but all of them have clearly recommended settings and are not expected to greatly affect the output. The first parameter is the number of principal components of the pathway enrichment vector space (computed from the matrix of pathway enrichment scores by cluster) used to identify a set of pathways to relate clusters when building the trajectory. This parameter is optimized based on a scree plot following principal component analysis done on the cluster pathway enrichment profiles. Tempora outputs a scree plot to help the user identify the elbow in the plot and select this parameter. As long as an elbow exists, the parameter range is very narrow for a user to select. A related, but not user definable parameter is the principal component (PC) loading threshold used to select pathways associated with each PC. This is set internally to >0.4 to select strong loadings, as recommended by “Stevens, J. P. (2012). Applied multivariate statistics for the social sciences”. A third parameter is the tolerance for leaving an edge undirected in the inferred trajectory when clusters have very similar temporal scores. The default is 0.01 (<1% difference) and we expect users will not frequently change this. From a user perspective, this mostly affects the visualization (edge style) of the inferred trajectory. Tempora uses all ARACNE edges and does not implement any thresholds to remove edges at this step. We have clarified these points in the methods.**

(5) Is Tempora sensitive to clustering results? To compare with Moncle2 and TSCAN, when using the same clustering results by Moncle2 and TSCAN, can Tempora still outperforms them?

**Response: We have input the same clustering results to all methods that we use in the benchmarking tests, except for CSHMM and Monocle 3, which do not take prior clustering as an input, and it does not change the results (see below). All benchmarking results for PAGA, Slingshot and TSCAN described in the main text are obtained with the same cluster input used in Tempora.**

**Trajectories of the human myoblast (HSMM) data set, inferred with Tempora cluster input (a-c) and without Tempora cluster input (d-f). Cells in the trajectories with Tempora cluster input are colored by their cluster assignments (a-c), while cells in the trajectories without cluster input (d-f) are labeled and colored by the milestones in the trajectories.**

**Trajectories of the murine cortex data set, inferred with Tempora cluster input (a-c) and without Tempora cluster input (d-f). Cells in the trajectories with Tempora cluster input are colored by their cluster assignments (a-c), while cells in the trajectories without cluster input (d-f) are labeled and colored by the milestones in the trajectories.**

**Trajectories of the murine cerebellar data set, inferred with Tempora cluster input (a-c) and without Tempora cluster input (d-f). Cells in the trajectories with Tempora cluster input are colored by their cluster assignments (a-c), while cells in the trajectories without cluster input (d-f) are labeled and colored by the milestones in the trajectories.**

Minor points:

(1) Since ARACNE is a key component of Tempora, please provide a detailed description of ARACNE in the supplementary.

**Response: We have described ARACNE in further detail in the methods section.**

(2) It is not clear how the time-dependent pathways are detected? For example, as in Fig 2b,c.

**Response: Tempora identifies time-dependent pathways by fitting a generalized additive model (GAM) on the pathway enrichment scores of each pathway across all clusters/time points and uses ANOVA to compare the fitted model with a null model of uniform pathway enrichment over time. We have added further description of how the time-dependent pathways are detected both in the main text and the Methods section.**

**Reviewer #3:**

Major comments:

**-**As the authors discuss, most current TI methods are integrated by the Dynverse group. Why then did the authors only compare their method to two of the oldest methods - Monocle and TSCAN - both of which didn't really perform that well in their benchmarking study? (10.1038/s41587-019-0071-9). The benchmarking really needs to be improved on several facets:

**-**More methods need to be included, which performed the best according to this benchmarking study and/or are the current state-of-the-art (e.g. Monocle 3 and PAGA)

**Response: This is an excellent point and we have now added three trajectory inference methods that score highly in Dynverse’s evaluation, including PAGA, Slingshot and Monocle 3, for additional comparison with Tempora (see Figure 5-7). We maintain TSCAN due to its use of clusters (like Tempora) and add CSHMM, a recent time series-based method (using time ordering information like Tempora).**

**-**Other methods that also use temporal information should be evaluated. Waddington-OT is an example of this (there are probably several others in the Dynverse libraries)

**Response: As mentioned in response to reviewer 1, we require a directed acyclic graph output from a TI method to compare to our gold standard benchmark trajectories (also represented as DAGs) and Waddington-OT does not provide this type of output. Instead, it provides a large network of temporal couplings between single cells. While we could imagine a few different ways to convert the temporal coupling network to a DAG or vice versa (e.g. minimum spanning tree), it was not clear that we could use these potentially arbitrary conversions not proposed by the original authors to create a fair method comparison. However, we have included new discussion about Waddington-OT’s advantages and complementary nature to Tempora. Additionally, we compared Tempora with CSHMM, a trajectory inference method for time-series scRNA-seq data based on a hidden Markov model (Lin and Bar-Joseph, Bioinformatics, 2019). Among the few time-series scRNA-seq trajectory inference methods we could find, we selected CSHMM because it is recently published, has functioning code, and has an output compatible with our benchmarking method. TASIC is similar to CSHMM (from the same authors), but is two years older and the authors report that it doesn’t scale well to more than a few time points. Two other methods that use time information as priors in the Dynverse library include SCUBA and MERLoT, but both failed to build at the time of this revision.**

**-**Tempora does not output pseudotime values for each of the cells, so I understand that using the "Cell positions" metric in the Dynverse study is difficult. Nontheless, it would be worthwhile to try it out, given that these authors of this benchmarking study also did apply it on methods that only output clusters

**Response: We did try comparing pseudotime to Tempora’s temporal ordering by correlating the average pseudotime of clusters calculated with different trajectory inference methods and Tempora’s temporal scores for each cluster. However, there is no gold standard benchmark to compare to to verify which predictions are correct, so we didn’t include this as part of our method comparison.**

**-**A comparison on 2 datasets is quite limited, especially given the sheer number of datasets available nowadays. I understand that time series information should be available, but there are also plenty of time series experiments, especially in the cell reprogramming field (e.g. several of the Treutlein lab's papers)

**Response: We have additionally evaluated Tempora on a data set of ~55,000 single cells from nine time points sampling the murine neural cerebellar development process (Figures 4, 7). We chose this data set because it is large, the lineages it contained are relatively complex, but well-studied, and the original publication included a ground-truth hand curated trajectory map that we could use to evaluate our prediction.**

**-**A benchmark on synthetic data can also be very valuable, I think both PROSSTT and the generator from Dynverse output time series data (or can be adapted in a way that they can).

**Response: We appreciate this suggestion. However, as we cannot easily simulate pathway information, which Tempora requires as input, it is not straightforward to benchmark Tempora on a synthetic data set. We are considering such development in future work.**

**-**How scalable is Tempora with increasing number of cells? I guess this is driven by the speed of the initial steps (pathway enrichment and clustering). How does this compare to other methods? (especially the most scalable ones from aforementioned benchmarking study)

**Response: This is a good question. Our analysis on the larger murine neural cerebellar development data set containing ~55,000 single cells and ~19,000 genes from nine time points (Figures 4, 7) shows that Tempora can scale to relatively large data sets. The main reason for this is the use of clusters by Tempora instead of single cells - this dramatically reduces the complexity of the task. Tempora and Monocle 3 are the only two methods that can be applied on this data set using a modern personal computer (MacBook Pro with 2.3 GHz Intel Core i9 processor and 16GB RAM) - Tempora takes on average 60 seconds and Monocle 3 takes 1700 seconds. Methods applied through the Dynverse wrapper, including PAGA, Slingshot and TSCAN, ran out of memory on this computer and necessitated running them on a larger machine with 120GB of memory where they completed successfully. We were never able to get CSHMM to finish running on this data even on a more powerful server and even after spending more than 180 hours of compute time. Running time for Tempora and Monocle 3 was measured from the start of the data import function (ImportSeuratObject() for Tempora and new_cell_data_set() for Monocle 3) to the end of the trajectory building functions (BuildTrajectory() for Tempora and learn_graph() for Monocle 3). We have mentioned these results in the discussion.**

**-**"Tempora assumes that user input includes an optimized clustering solution for their data." In my experience, the clustering is the step that has the largest influence on the quality of an inferred trajectory. If I understand correctly, the authors used the same clustering strategy for both datasets, i.e. using scClustViz and selecting the # of clustering as described in the methods. Do the authors suggest that the users always use this strategy? How can we be sure that this clustering is not the reason why Tempora performs better than Monocle and TSCAN? Why didn't the authors provide this clustering to either of these methods, or alternatively use the clustering present in either of these methods for Tempora?

**Analysis: We suggest that users use their own clustering and annotation strategy, but scClustViz presents a good starting point for users looking for a recommended strategy. We have now input Tempora’s clustering to all methods that we use in the benchmarking tests, except for CSHMM, which does not take prior clustering as an input, and it does not change the results (please see comparisons in our response to Reviewer 2).**

**-”**To calculate the mismatch score between a pair of graphs, we first label each cluster in the inferred trajectory with the cell type(s) it contains, based on expression of a set of wellknown marker genes." Does this mean that this was done separately for the clusters obtained by Tempora, and the clusters obtained by Monocle and TSCAN? This looks like a very arbitrary way to label clusters, and can easily add a bias into this benchmark which is supposed to be objective. Why didn't the authors use a method that can label cells/clusters automatically? (many of them are available, some of which allow you to provide a set of markers for each cell type). This would make the analysis more objective.

**Response: Sorry for the confusion. All data sets were automatically annotated with GSVA using cell type markers published with the scRNA-seq data (S1 Table). Annotation for each data set was done once per data set and used for all analyses. We updated the text to clarify this. In case users don’t already have annotations, we added a convenience feature to Tempora to help authors annotate clusters based on GSVA, recommended via benchmarking in Diaz-Mejia et al. (https://f1000research.com/articles/8-296).**

**-**How would the accuracy score of Tempora change if you would determine the direction of the edges in the same way as you did for Monocle and TSCAN (as described in the methods)? The main selling-point of Tempora is that it uses time information, so this is an important experiment to check whether the temporal information adds something (compared to determining the direction using marker genes)

**Response: We performed this analysis and added it to the manuscript. Determining directions with marker genes does not lead to any changes in the HSMM trajectory, as all clusters are connected to the root myoblast cluster. However, for more complex trajectories in which there is more than one root state, including the murine cortex and cerebellum data sets, Tempora’s performance decreases when directions are determined without time as edges not connected to the roots become bidirectional. These results are presented in Supplementary Figure 11.**

**-**It seems to me that the "similarity threshold" is an important parameter, that depends on the dataset. What value did the authors choose for the datasets? Was this optimized? If so, how can we be sure that the benchmark is not biased towards Tempora given that the parameters were not optimized for Monocle and TSCAN?

**Response: The similarity threshold identifies clusters that have highly similar temporal scores (less than 1% difference), in which case we are not confident to assign an edge direction based on time ordering. These edges are visualized as dashed lines in the output trajectory and counted as bidirectional edges for benchmark comparisons. The 1% similarity threshold is the default parameter in the code and used in all results presented in the paper. The parameter was optimized over our three benchmark data sets, but we recognize that it could be revisited after Tempora is applied more generally. Only the mouse cortex data set was observed to have such clusters with similar time scores (one dashed line in Figure 3a). This parameter is not expected to have much effect on Tempora’s performance. We have now clarified the meaning of this parameter in the text.**

Minor comments:

**-”**The central concept used by most of these methods to infer temporal cell trajectories is that cells that are close to each other in time (e.g. cells that are closely related to each other via differentiation) will have similar transcriptomes. However, sometimes actual time point information is available to use for this purpose, in which case it should be used directly." This section of the Author Summary is confusing to me. What is meant by "time" here, pseudotime or real-life time? Most trajectory inference methods do not assume that cells are close to each other in real-life time (i.e. snapshot data). I think it should be clearly defined how cells can be related in time, i.e. if their differentiation process happens in a synchronous manner (i.e. time-series data).

**Response: We apologize for the confusion. We have clarified the wording of the Author Summary and remove the part about time. We meant that cells that are closely related in a lineage will have similar transcriptomes.**

**-**It's a bit weird that Monocle v1 (ICA/MST) is discussed in detail in the introduction, but then Monocle v2 is used in the evaluation. Also, it should always be clearly stated which of these two methods are meant, because the name "Monocle" is used for both v1 and v2 throughout the manuscript (especially given that v3 also exists, which can be even more confusing for people new to the field).

**Response: We have clarified our description of Monocle v1 and v3 in the introduction and throughout the text. Monocle v3 is now used in the benchmarking tests.**
