# Supplementary material for: Tempora: Cell trajectory inference using time-series single-cell RNA sequencing data
Source: PLoS Comput Biol. 2020 Sep 9;16(9):e1008205. doi: 10.1371/journal.pcbi.1008205 (PMC7505465; doi:10.1371/journal.pcbi.1008205)
Supplement: Attachment — Submitted filename: Tempora_Response_to_Reviewers_Round2.docx [file pcbi.1008205.s015.docx]

Dear Editor,

Thank you for the opportunity to submit a revised draft of our manuscript titled “Tempora: cell trajectory inference using time-series single-cell RNA sequencing data” to PLOS Computational Biology. We have fully addressed the few remaining minor concerns and comments as detailed below.

**Reviewer #1:**

Executive Summary:

In this revision, the authors have expanded on the number of trajectory inference methods compared and also included a larger murine cerebellar dataset for benchmarking. The authors have addressed my previous concerns. I have the following set of minor comments:

1. While the authors have included new analyses highlighting the importance of batch correction prior to trajectory inference, it remains unclear whether the inferior performance noted for Monocle for example (and also the additional methods) could be attributed to inferior batch correction rather than the trajectory inference itself. Presumably, a minimum spanning tree could be constructed on the Harmony-corrected PCs, so a comparison based on the same batch-correction across trajectory inference methods should be achievable.

**Response: To compare the effect of Harmony batch correction on Monocle 3’s performance, we replaced the principal components calculated through PCA by Monocle 3 with Harmony-corrected PCs, then constructed trajectories based on the recommended workflow. For all three validation data sets, Harmony-corrected PC input to Monocle 3 does not lead to improvement in accuracy scores or mismatch scores of the inferred trajectories (please see below). Therefore, for Monocle 3, it is unlikely that inferior batch correction accounts for its inferior performance compared to Tempora.**

**An update of figure S9 showing the effect of two batch effect correction approaches (Batchelor and Harmony) on Monocle 3 performance. Monocle 3 trajectories and batch correction comparison of a,b,c. HSMM, d,e,f. murine cerebral cortex and g,h,i. murine cerebellar data. j-k. Performance evaluation of Monocle 3 on the benchmarking data with and without batch effect correction.**

**We were not able to force CSHMM or methods applied through Dynverse (PAGA, Slingshot and TSCAN) to run on Harmony PCs. While it is theoretically possible to achieve this, it would require major changes in the algorithm implementations. We added a note to the manuscript that this is still an open question and that future work in this field should evaluate the effects of batch correction on trajectory inference.**

2. With the new larger murine cerebellar dataset, the authors now note that "when applied on the ~19,000 gene x ~55,000 cell gene mouse cerebellar development expression matrix, Tempora completes in an average of 60 seconds, while Monocle 3 takes 1700 seconds on a modern personal computer." While this is a notable improvement in computational efficiency, does Tempora scale linearly with the number of genes or cells? Or will the runtime growth be exponential with the number of clusters? It may be useful for readers as the size and complexity of single-cell RNA-seq datasets increases.

**Response: Thank you for this comment. To evaluate whether Tempora scales linearly with the number of genes or cells, we downsampled the mouse cortex data set, which contains ~15,700 genes x ~6,000 cells, to different numbers of cells or genes, and measured the runtime of Tempora on the subset data. Results from this analysis (below) show that Tempora scales linearly with both number of genes and cells. We observe the same results with the larger mouse cerebellum data (S12 Fig). We have added this information to our manuscript discussion and supplementary results.**

**Reviewer #2:** The authors have done an excellent job in revision of the manucript and all my concerns have been addressed. I have one comment for the authors. The dimentionality reduction is very important for the downstream analysis of time series scRNA-seq data, but few study has realized it. I hope the author can take a look at the TSEE algorithm (BMC Genomics 2019: <https://doi.org/10.1186/s12864-019-5477-8>), the first dimentionality reduction algorithm that can incooperate the time stage information of time series scRNA-seq data. It outperforms existing methods (e.g. PCA, t-SNE) in preserving local and global structures as well as enhancing the temporal resolution of samples. It can uncover the subtle gene expression patterns, facilitating further downstream analysis.

**Response: Thank you for this suggestion. The TSEE algorithm definitely looks useful for time-series scRNAseq data and we will explore incorporating it as an alternative dimensionality reduction method in a future release of Tempora. We have now mentioned and cited TSEE in our manuscript.**

**Reviewer #3:** The authors have fulfilled all my concerns, and by doing that, the authors have improved the methodology and scientific soundness of their study. I have no further concerns.

**Response: Thank you for the very helpful review of our manuscript.**
